# Plant and prokaryotic TIR domains generate distinct cyclic ADPR NADase products

**DOI:** 10.1126/sciadv.ade8487

**Published:** 2023-03-17

**Authors:** Adam M. Bayless, Sisi Chen, Sam C. Ogden, Xiaoyan Xu, John D. Sidda, Mohammad K. Manik, Sulin Li, Bostjan Kobe, Thomas Ve, Lijiang Song, Murray Grant, Li Wan, Marc T. Nishimura

**Affiliations:** ^1^ Department of Biology, Colorado State University, Fort Collins, CO 80523, USA.; ^2^ National Key Laboratory of Plant Molecular Genetics, Center for Excellence in Molecular Plant Sciences, Institute of Plant Physiology and Ecology, Chinese Academy of Sciences, Shanghai 200032, China.; ^3^ Cell and Molecular Biology Graduate Program, Colorado State University, Fort Collins, CO 80523, USA.; ^4^ School of Life Sciences, University of Warwick, Coventry CV47AL, UK.; ^5^ The University of Queensland, School of Chemistry and Molecular Biosciences, Australian Infectious Diseases Research Centre and Institute for Molecular Bioscience, Brisbane, QLD 4072, Australia.; ^6^ Institute for Glycomics, Griffith University, Southport, QLD 4222, Australia.

## Abstract

Toll/interleukin-1 receptor (TIR) domain proteins function in cell death and immunity. In plants and bacteria, TIR domains are often enzymes that produce isomers of cyclic adenosine 5′-diphosphate–ribose (cADPR) as putative immune signaling molecules. The identity and functional conservation of cADPR isomer signals is unclear. A previous report found that a plant TIR could cross-activate the prokaryotic Thoeris TIR–immune system, suggesting the conservation of plant and prokaryotic TIR-immune signals. Here, we generate autoactive Thoeris TIRs and test the converse hypothesis: Do prokaryotic Thoeris TIRs also cross-activate plant TIR immunity? Using in planta and in vitro assays, we find that Thoeris and plant TIRs generate overlapping sets of cADPR isomers and further clarify how plant and Thoeris TIRs activate the Thoeris system via producing 3′cADPR. This study demonstrates that the TIR signaling requirements for plant and prokaryotic immune systems are distinct and that TIRs across kingdoms generate a diversity of small-molecule products.

## Teaser

Immune pathways of plants and prokaryotes are activated by distinct TIR-generated metabolites.

## INTRODUCTION

Globally, plant pathogens are estimated to diminish crop yields by over 15% each year and are a major threat to food security (*1*, *2*). Understanding the mechanistic details of the plant immune system is a critical requirement for rationally engineering disease resistance. Unlike animals, plants do not have adaptive immune systems and must encode expansive repertoires of cell-autonomous innate immune receptors to defend against pathogens. Toll/interleukin-1 receptor (TIR) domains are encoded by plants, animals, and prokaryotes and typically function in cell death and innate immune pathways (*3*–*6*). The TIR domains of animal Toll-like receptors transduce immune signals via direct protein-protein interactions (*6*). The discovery that the human TIR-containing protein, sterile alpha and TIR-motif containing 1 (SARM1), executes axonal degeneration via NAD^+^ (nicotinamide adenine dinucleotide)–hydrolase activity was pivotal to understanding TIR immunity in plants and prokaryotes (*3*, *7*, *8*). TIR-immune proteins of both plants and prokaryotes are now known to be enzymes that consume and/or modify nucleotides (including NAD^+^) or nucleic acids, and this enzymatic function is required for immune signaling across the tree of life (*3*, *9*–*14*). The number and type of identified small molecules produced by enzymatic TIRs are expanding rapidly (*3*, *4*, *15*). Recently, it was reported that the TIR-immune signals of plants and of a prokaryotic antiphage immune system, Thoeris, might be conserved (*16*). The identity of the Thoeris TIR [ThoerisB (ThsB)]–produced immune signal is unknown, and it is unclear if this prokaryotic immune signal might cross-activate plant TIR–immune pathways. Deciphering the identity and immune outputs of TIR-generated metabolites is key to understanding and engineering TIR signaling pathways.

Plants initially sense potential pathogens via extracellular receptors that recognize conserved microbe-associated molecular patterns (MAMPs) (*17*). Upon binding MAMPs, these receptors trigger intracellular signaling cascades that activate an initial immune response known as PTI (pattern-triggered immunity) (*17*). However, adapted pathogens can disarm host PTI responses via delivering virulence factors or “effectors,” which manipulate host defense responses and/or physiology (*18*). Accordingly, plants have evolved a second layer of intracellular disease resistance proteins known as “R” proteins, which recognize effectors or their activities and signal a rapid immune response termed ETI (effector-triggered immunity) (*17*). ETI often results in host cell death (the hypersensitive response), and plant “R proteins” generally contain N-terminal TIR or CC (coiled-coil) domains coupled to a nucleotide-binding site leucine-rich repeat (LRR) (NLR) chassis (*17*). The C-terminal NLR domain confers effector recognition and provides an oligomerization chassis, which promotes N-terminal TIR or CC domain activation and immune signaling (*17*). TIR-NLR proteins are encoded by dicots, gymnosperms, and even single cellular algae (*4*, *5*, *19*). Curiously, monocots encode TIR-only (but not TIR-NLR) proteins that can cross-activate dicot TIR-immune pathways, although potential TIR immunity within monocots remains less well characterized (*12*).

Similar to the immune TIRs of plants, the prokaryotic ThsB TIR generates immune signals using enzymatic activities (*16*). However, Thoeris defense requires only a single downstream mediator/executioner to initiate cell death—the sirtuin2 (SIR2)–type NADase, ThsA. Upon binding unknown ThsB-derived immune signals via a C-terminal Smf/DprA-LOG (SLOG) domain, ThsA causes host cell death by rapidly depleting cellular NAD^+^, thereby halting phage replication (*16*). Apart from roles in immunity, certain microbial TIR-domain NADases have even been co-opted as virulence factors (*20*, *21*). By contrast, plant TIR–immune signals must be relayed by several mediators: EDS1 (enhanced disease susceptibility 1) family members and, subsequently, the helper NLRs, NRG1 (N requirement gene 1) or ADR1 (activated disease resistance 1) (*3*, *5*). EDS1 is a lipase-like protein that forms exclusive heterodimers with EDS1 family members phytoalexin-deficient 4 or senescence-associated gene 101, and these heterodimers were recently shown to bind the TIR-generated signals adenosine 5′-diphosphate (ADP)–ribosylated adenosine 5′-triphosphate (ATP) (ADPr-ATP), ADP-ribosylated ADP (ADPr-ADP) and phosphoribosyl-5’–adenosine 5′-monophosphate (AMP)/ADP (pRib-AMP/ADP) (*3*, *4*, *9*, *10*). Upon interacting with EDS1 heterodimers, the NRG1 or ADR1 helper NLRs oligomerize into Ca^2+^-permeable pores and transduce the initial TIR-immune signal into hypersensitive cell death (HR) and/or transcriptional defense programs (*22*–*24*). Certain plant TIR domains were recently reported to bind and hydrolyze DNA or RNA substrates to generate 2′,3′ cyclic AMP/guanosine 5′-monophosphate, although the EDS1 dependence and/or putative immunological role of TIR-generated 2′,3′cNMPs (cyclic nucleotide monophosphates) is unclear (*11*, *25*).

Certain mechanistic features of enzymatic TIRs are conserved even across very distant phyla (*3*, *12*, *26*). For instance, all examined prokaryotic and eukaryotic TIRs require a conserved glutamate (E) residue for catalysis (*3*, *7*, *13*, *14*, *16*). In addition, enzymatic TIRs contain a flexible loop, termed the BB-loop, which is laid over the catalytic pocket and has been proposed to regulate substrate access (*14*, *27*–*29*). Enzymatic TIR domains also require self-association via TIR-TIR interfaces to engage in catalysis (*12*–*14*, *29*). Cryo–electron microscopy (cryo-EM) studies have provided key insights into how oligomerization and self-association promote the activation of plant TIR-NLRs and CC-NLRs (*27*, *28*, *30*, *31*). For example, the structure of the pentameric ZAR1 [hypersensitive response and pathogenicity-dependent outer protein (Hop)Z-activated resistance 1] “resistosome” reveals that pathogen effectors induce the assembly of CC-NLRs into ring-shaped Ca^2+^-ion channels (*30*, *32*). Similarly, the structures of the activated TIR-NLRs, RPP1 (recognition of *Peronospora parasitica* 1) and Roq1 (recognition of XopQ1, Xanthomonas outer protein Q1), indicate that effector activities also induce TIR-NLRs to assemble into tetramers, thereby engaging their enzymatic cores (*27*, *28*). The crystal structure of the Thoeris ThsB TIR protein reveals a core-TIR domain, followed by a small C-terminal β sheet domain; the structure also suggests that ThsB could form dimers (*33*). Bacteriophage triggers the TIR-NADase activity of ThsB, although the mechanistic details of activation and potential ThsB-ThsB oligomerization requirements are unknown (*16*, *33*). Plants also encode TIR domains that lack canonical NLR architecture, including TIR-NB, TIR-only proteins, and X-TN-X proteins (*34*–*36*). A recent study found that a plant TIR–only protein, Response to HopBA1 (RBA1), can self-associate and perform enzymatic functions as linear filaments on nucleic acids (*11*). The atypical X-TN-X TIR architecture may activate cell death independently of the TIR pathway mediator, EDS1 (*36*).

As noted, plant and prokaryotic immune TIRs, as well as human SARM1, require enzymatic activity for their functions in immunity and axonal cell death, respectively. SARM1 NADase activity produces ADP-ribose (ADPR) and canonical cyclic ADPR (cADPR), which are both secondary messengers that can trigger intracellular Ca^2+^ signaling cascades within animal cells (*15*, *37*, *38*). The TIR domains of plants and prokaryotes produce noncanonical cADPR isomers from NAD^+^. These NADase products, originally called “variant cADPR” (v-cADPR), were not differentiated by chromatography in early studies; however, subsequent studies have revealed that there are at least two isoforms (3′cADPR or 2′cADPR) (*3*, *12*, *14*, *20*, *26*, *39*). These analytic difficulties have confounded strong interpretation of any in planta function for cADPR isomers (*12*, *26*). While v-cADPR was used as a biomarker of plant TIR pathway activation by pathogens, a later study reported that v-cADPR generation by a prokaryotic TIR, AbTir (from human pathogen *Acinetobacter baumanii*), was insufficient to stimulate EDS1-mediated plant HR (*40*). 3′cADPR is the NADase product of the HopAM1 TIR protein—a virulence factor encoded by the plant pathogen *Pseudomonas syringae* pathovar *tomato* DC3000 (*20*). Potential conservation among plant TIR and prokaryotic ThsB TIR–derived immune signals remains unclear (*16*, *41*). Here, we examined whether the NADase-derived signals from the Thoeris immune TIR, ThsB, are compatible with plant TIR immune pathways to clarify the diversity and function of TIR enzymatic products across the tree of life.

## RESULTS

### Prokaryotic TIR NADases cause cytotoxicity independent of the plant TIR–signal mediator, EDS1

**Fig. 1.**
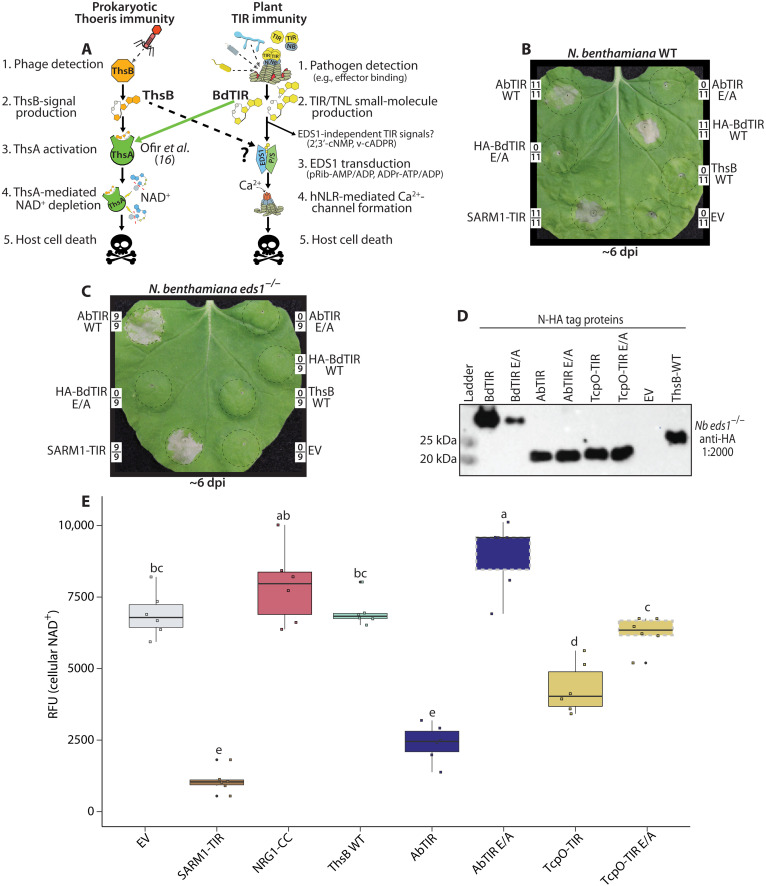
Prokaryotic TIRs can trigger in planta cytotoxicity independently of EDS1. (**A**) Schematic of prokaryotic Thoeris immunity and plant TIR–immune signaling. In both systems, TIRs sense pathogens and signal immune outputs via NADase activities. Plant TIRs generate multiple metabolites, some of which are known to activate EDS1-dependent outputs (pRib-AMP/ADP and ADPr-ATP/ADP), while potential EDS1-independent outputs remain unknown. BdTIR stimulates both EDS1 and Thoeris outputs; does ThsB stimulate EDS1 outputs (see dashed arrow)? (**B** and **C**) *Nb* WT or *eds1*^−/−^ leaves shown approximately 6 dpi with constructs delivering AbTIR, HA-BdTIR, ThsB, or EV [empty vector control; 35S: green fluorescent protein (GFP)]. E/A refers to TIRs containing alanine substitutions at the conserved catalytic glutamate (E) residue. Framed numbers denote leaf replicates per set. (**D**) Anti-HA immunoblot of N-HA–tagged BdTIR, AbTIR, ThsB, or TcpO-TIR proteins harvested from *Nb eds1*^−/−^ leaves at ~40 hours postinfiltration (hpi). (**E**) Fluorescent NAD^+^ detection assay in *Nb eds1*^−/−^ leaves expressing different TIR constructs. NAD^+^ assays were performed at 40 hpi. RFU, relative fluorescence units. NRG1-CC is the CC domain of the CC-NLR protein, NRG1. AbTIR (core TIR from AbTir) residues 157 to 292, TcpO-TIR residues 204 to 341, and SAM_SARM1-TIR residues 478 to 724. Catalytic glutamate (E residue) mutants of TIRs were outlined in dashed gray boxes; AbTIR (E208), BdTIR (E127), and TcpO-TIR (E279). Similar experiments were performed at least three times. Statistical analyses: One-way analysis of variance (ANOVA) and Turkey honestly significant difference (HSD) with CLD (compact letter display) of significance classes. Overlapping letters are ns (nonsignificant) difference (*P* > 0.05), while separate letter class indicates *P* < 0.05 or better.

Plant TIR–enzymatic activities are required to signal EDS1-mediated HR (*12*, *13*). As plant BdTIR (from *Brachypodium distachyon*) cross-activated ThsA, the mediator of Thoeris immunity (*16*), we tested whether the ThsB immune TIR (of *Bacillus cereus* MSX D-12) could generate signals that cross-activated EDS1 in planta (see schematic diagram in Fig. 1A). Accordingly, we synthesized and expressed codon-optimized ThsB in the plant *Nicotiana benthamiana* (*Nb*) and monitored EDS1-mediated HR (Fig. 1, B and C). We expressed plant BdTIR as a positive control for HR and the human TIR NADase, SARM1, as an EDS1-independent cell death inducer (via NAD^+^ depletion) (*12*). In addition, we expressed the core TIR domain of AbTir (core-TIR denoted as “AbTIR”), a nonimmune TIR from the pathogenic bacterium *Acinetobacter baumannii*, which generates 2′cADPR (*14*, *40*). BdTIR expression triggered HR in wild-type (WT) *Nb*, but not in plants lacking *EDS1* (*Nb eds1*^−/−^), while SARM1-TIR elicited cell death in both (Fig. 1, B and C). Similar to SARM1-TIR, AbTIR triggered chlorotic cell death in both WT and *eds1^−/−^* plants, while ThsB caused no apparent phenotype (Fig. 1, B and C). We also assayed another v-cADPR–producing TIR domain from the Archaea *Methanobrevibacter olleyae* (TcpO-TIR), which likewise caused mild chlorosis independent of EDS1 (fig. S1) (*12*, *26*). Chlorotic cell death by AbTIR or TcpO-TIR required the conserved TIR domain catalytic glutamate (E), indicating that while these TIRs are enzymatically active, neither produces EDS1-activating signals (Fig. 1, B and C). We also observed that elevating BdTIR expression [via adding Omega translational enhancers (*42*)] could similarly cause a slow chlorotic cell death independent of EDS1 (fig. S1). AbTIR, TcpO-TIR, and ThsB were phenotyped without epitope tags, while N-terminal hemagglutinin (N-HA) tags were used to confirm expression via immunoblot (Fig. 1, C and D). Structural models indicating the conserved TIR domain structure and catalytic glutamate (E) of AbTir and TcpO are provided in fig. S1 (A and B). Similar to plant TIR-NLR immune receptors, ThsB NADase activities are triggered by pathogens (i.e., bacteriophages), and the lack of an apparent phenotype suggested that ThsB might be inactive in the absence of this trigger (*16*, *33*).

SARM1-mediated cell death in neurons and plants correlates with a strong depletion of NAD^+^ (*7*, *12*, *13*). Thus, we used a fluorescence-based NAD^+^ assay to determine whether AbTIR, TcpO-TIR, or ThsB depleted NAD^+^ in planta (Fig. 1E). SARM1-TIR was included as a positive control for NAD^+^ depletion (Fig. 1E). AbTIR and TcpO-TIR both reduced cellular NAD^+^, suggesting that the EDS1-independent toxicity in Fig. 1B may be explained by perturbation of NAD^+^ homeostasis, similar to SARM1-TIR. NRG1-CC causes rapid cell death via Ca^2+^-channel formation and serves as a control to indicate that plant cell death per se does not drive NAD^+^ depletion (Fig. 1E). As above, ThsB elicited no apparent NAD^+^ depletion, despite the abundant protein accumulation (Fig. 1D). To try to find an active ThsB allele, we assayed 11 additional ThsB orthologs encoded by other bacterial species; none caused apparent phenotypes (fig. S2). Together, these results suggest that while v-cADPR–producing AbTIR and TcpO-TIR do not trigger EDS1-dependent immunity, highly active TIR NADases can be cytotoxic via cellular NAD^+^ depletion. Neither TcpO nor AbTir has known roles in host immune signaling. Thus, we refocused on generating ThsB enzymatic activity in planta, to assess the conservation of plant and prokaryotic TIR-immune signals.

### Replacement of a ThsB loop region promotes autoactivity and stimulation of the Thoeris mediator, ThsA

Thoeris immunity (*B. cereus* MSX D12) is initiated by phage detection, indicating that ThsB signaling is inactive before infection (*16*). Hence, we hypothesized that an autoactive ThsB NADase might be created by removing or modifying negative regulatory regions within ThsB. An autoactive ThsB should hydrolyze NAD^+^ and generate cADPR isomers, which stimulate the Thoeris partner, ThsA. In addition, if the TIR-immune signals of plants and the Thoeris system are conserved, then an autoactive ThsB might stimulate EDS1-mediated HR (see Fig. 1A model).

**Fig. 2.**
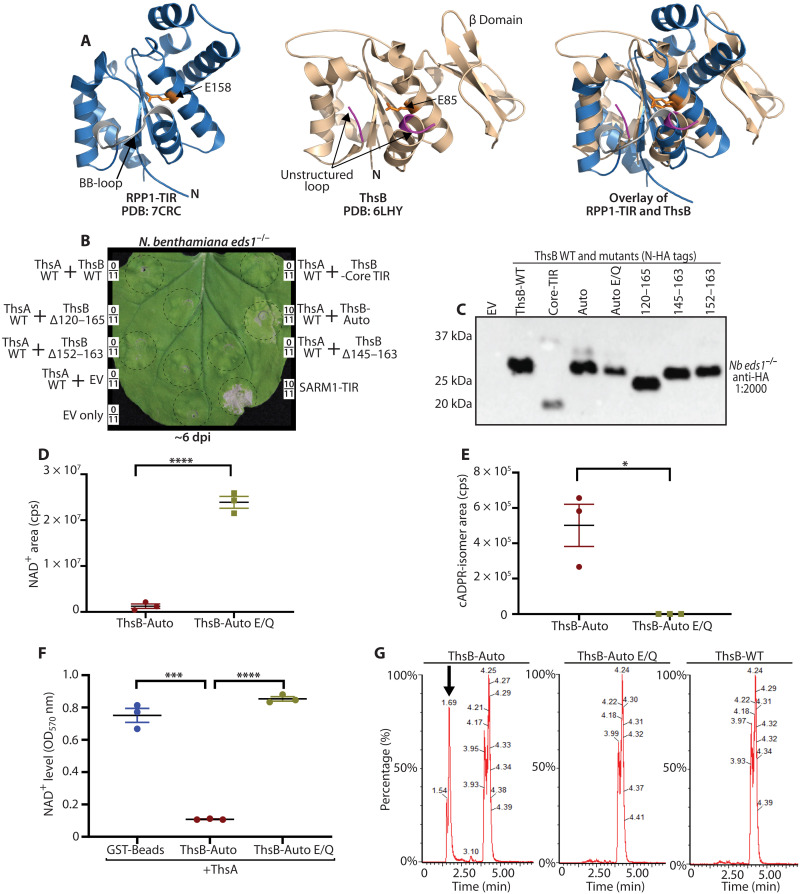
Mutagenesis of a ThsB loop region promotes NADase autoactivity, cADPR-isomer production, and stimulation of ThsA-mediated cytotoxicity. (**A**) Left: Cryo-EM structure of activated-state TIR domain from plant TIR-NLR, RPP1 [Protein Data Bank (PDB): 7CRC]. Center: Crystal structure ThsB (PDB: 6LHY). Right: Overlay of activated RPP1-TIR with ThsB. The RPP1 BB-loop is colored gray, ThsB loop region is shown in purple, and catalytic glutamates are shown in orange. N, N terminus. (**B**) *Nb eds1*^−/−^ leaf ~6 days postagroinfiltration (dpi) with constructs coexpressing ThsA with WT ThsB, ThsB-mutants, or EV (35S: GFP). ThsA and ThsB were coexpressed at an individual optical density (OD) of 0.40. Positive and negative control SARM1-TIR or EV, respectively, expressed individually at an OD of 0.80. Framed numbers denote leaf replicates per set. (**C**) Anti-HA immunoblot of N-HA–tagged ThsB variants were transiently expressed in *Nb eds1*^−/−^ leaves and harvested ~40 hpi. (**D** and **E**) In vitro NAD^+^ consumption by recombinant ThsB-Auto or ThsB-Auto E85Q (catalytic mutant) and detection of cADPR-isomer via LC-MS. (**F**) In vitro ThsA NADase stimulation by recombinant ThsB-Auto or ThsB-Auto E85Q (catalytic mutant) or glutathione *S*-transferase (GST)–laden beads alone. (**G**) LC-MS traces for cADPR isomers (MW 542) in *Nb eds1*^−/−^ leaves transiently expressing ThsB-Auto, ThsB-Auto E85Q, or WT ThsB; leaves sampled ~40 hpi. Arrow denotes ThsB-Auto–produced cADPR isomers. Similar experiments were performed at least three times. Statistical analyses: One-way ANOVA and Turkey HSD. *P < 0.05, **P < 0.01, ***P < 0.005, and ****P < 0.0005.

A previous study by Ka *et al.* (*33*) determined the crystal structures of ThsB and the mediator, ThsA. To gain insights into potential ThsB regulatory regions, we modeled ThsB onto the structure of an activated-state plant TIR-NLR, RPP1 (Fig. 2A) (*28*, *33*). The activated structures of RPP1 and ROQ1 TIR domains indicate that movement of a loop (the “BB-loop”) near the catalytic glutamate may be important for allowing substrate access and/or catalysis (Fig. 2A) (*28*). We noted that ThsB also has a putative BB-loop in this position, although it is not resolved in the ThsB crystal structure. Unlike plant TIR domains, ThsB contains a large C-terminal β sheet following the TIR-domain (Fig. 2A). Thus, we hypothesized that this loop near the catalytic pocket and/or the C-terminal β sheet might affect ThsB NADase activation. Accordingly, we generated and examined five different ThsB variants for autoactivity: a modified “loop” region and four progressive deletions of the “β sheet” domain (Fig. 2 and fig. S3). Briefly, the loop variant substituted loop residues with glycines, while each C-terminal variant removed the denoted residues and added a short glycine linker. These five ThsB variants were named: “Auto” (loop region), “Core-TIR,” “120 to 165,” “145 to 163,” and “152 to 163.” Figure S3 maps each of these modifications onto the ThsB crystal structure.

Because the TIR domains of AbTir and SARM1 deplete cellular NAD^+^ and cause chlorotic cell death independent of EDS1 (Fig. 1B), we reasoned that ThsB activation of ThsA, an SIR2-type NADase, might cause similar chlorosis and cytotoxicity. Thus, we screened each ThsB variant for autoactivity by coexpressing it with ThsA and monitoring for chlorosis (Fig. 2B). ThsA coexpression with WT ThsB, or any C-terminal deletion, did not trigger chlorosis (Fig. 2B). An anti-HA immunoblot confirmed accumulation for all ThsB variants (Fig. 2C). When ThsA was coexpressed with the ThsB-loop deletion, termed Auto, a mild chlorotic cell death appeared ~4 to 5 dpi (days postagroinfiltration) (Fig. 2B). To attempt to increase the activity of ThsB-Auto, we generated additional mutants in the BB-loop (fig. S3, C and D). While these additional BB-loop mutants were also autoactive, neither had appreciably stronger phenotypes than the original ThsB-Auto mutant. We also examined the effects of similarly altering the BB-loop of plant TIR proteins; however, we found that this caused a loss of EDS1 signaling function (fig. S3, E to G).

We next determined whether ThsB-Auto produced cADPR isomers from NAD^+^ (Fig. 2, D to G). Using recombinant ThsB-Auto protein, we performed in vitro NADase assays and LC-MS (liquid chromatography–mass spectrometry) and detected the consumption of NAD^+^ and cADPR isomer generation (Fig. 2, D and E). Furthermore, the NADase products of ThsB-Auto could stimulate NAD^+^ consumption by ThsA in vitro (Fig. 2F). We also used LC-MS and detected cADPR isomer production by ThsB-Auto in planta (Fig. 2G). cADPR isomer generation by ThsB required the catalytic glutamate (E85), as did in vitro stimulation of ThsA (Fig. 2, D to G). Together, these findings indicate that the TIR NADase functions of ThsB can be activated by modifying a portion of its BB-loop and suggest that this loop may play a part in regulating Thoeris signaling. The NAD^+^ breakdown products from ThsB-Auto stimulated ThsA activity in vitro, in addition to stimulating in planta ThsA cytotoxicity (Fig. 2B).

### ThsB-Auto requires the TIR domain catalytic glutamate to stimulate ThsA-mediated NAD^+^ depletion and cytotoxicity

**Fig. 3.**
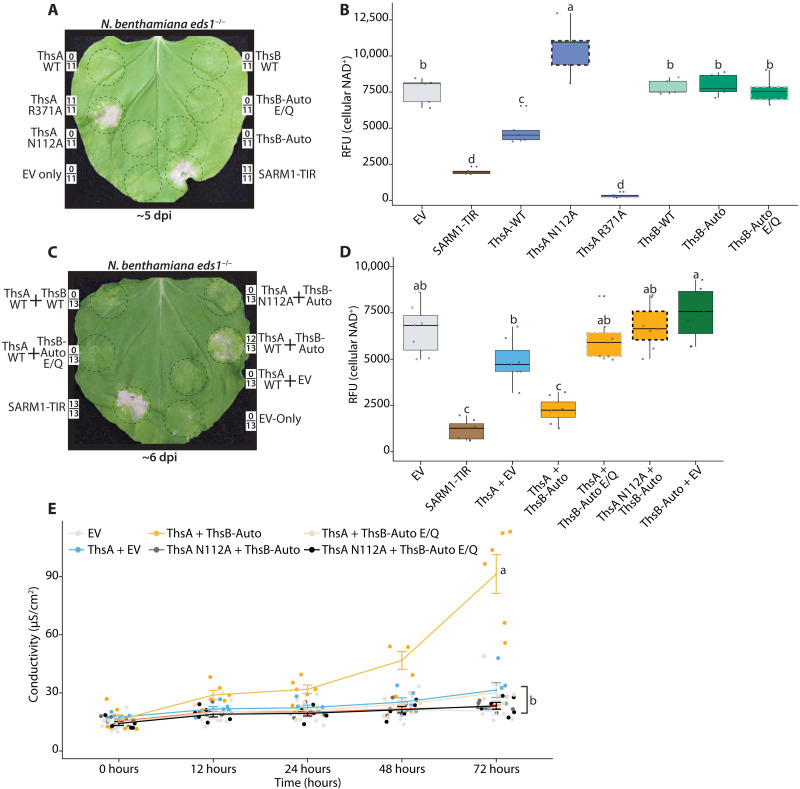
ThsB-Auto requires TIR-NADase functions to stimulate ThsA-mediated NAD^+^ depletion and cytotoxicity. (**A**) *Nb eds1*^−/−^ leaf ~5 dpi with constructs expressing WT ThsA or ThsB, ThsA, and ThsB variants, or SARM1-TIR or EV (35S: GFP) controls. ThsA N112A lacks SIR2-type NADase activity; ThsA R371A has an altered SLOG-motif, and ThsB E85Q lacks TIR domain catalytic activity. Constructs expressed at an OD of 0.80. Framed numbers denote leaf replicates per set. (**B**) Fluorescent NAD^+^ detection assay in *Nb eds1*^−/−^ leaves expressing various ThsA or ThsB constructs. NADase assays were performed at 40 hpi. (**C**) *Nb eds1*^−/−^ leaf coexpressing ThsA or ThsA N112A with ThsB-Auto, ThsB-Auto E/Q, or EV, shown ~6 dpi. ThsA and ThsB coexpressed at an OD of 0.40; positive and negative control SARM1-TIR and EV expressed at an OD of 0.80. Catalytic TIR (E/A) mutants outlined in dashedgray; ThsA N112A outlined in dashedblack. (**D**) Fluorescent NAD^+^ detection assay in *Nb eds1*^−/−^ leaves coexpressing ThsA and ThsB combinations. NAD^+^ assays performed at 40 hpi. Catalytic glutamate mutants are outlined in dashedgray; inactive ThsA N112A is outlined in dashedblack. (**E**) Ion leakage assay in *Nb eds1*^−/−^ leaves coexpressing ThsA and ThsB combinations. Leaf discs were collected ~72 hpi, and statistical analyses were performed for final time point. Similar experiments were performed at least three times. Statistical analyses: One-way ANOVA and Turkey HSD with CLD of significance classes. Overlapping letters are ns difference (*P* > 0.05), while separate letter class indicates *P* < 0.05 or better.

During Thoeris immunity, stimulated ThsA drives host cell death via NAD^+^ depletion and restricts phage replication (*16*). To validate ThsA stimulation by ThsB-Auto in planta, we expressed ThsA and ThsB alone, or together, and examined cellular NAD^+^ levels and cytotoxicity in *Nb eds1*^−/−^ plants (Fig. 3, A to E). As controls, we included previously described ThsA N112A, which lacks SIR2-type NADase activity, and ThsA R371A, which is reportedly impaired in binding activating cADPR isomer (*14*, *16*, *33*). Figure S4 maps N112 and R371 onto the ThsA crystal structure (*33*). When expressed alone, neither WT ThsB, ThsB-Auto, nor ThsB-Auto E85Q diminished NAD^+^ or caused cytotoxicity similar to positive control SARM1-TIR (Fig. 3, A and B). ThsA alone has been reported to have some constitutive but weak background NADase activity in vitro (*14*, *16*, *33*). Consistent with this, ThsA mildly reduced NAD^+^ relative to empty vector or NADase-null ThsA N112A controls in planta (Fig. 3B). NAD^+^ consumption by unstimulated ThsA was not enough to trigger qualitative or quantitative cytotoxicity (Fig. 3, A, C, and E). Unexpectedly, ThsA R371A displayed enhanced NAD^+^ depletion compared to WT ThsA and accordingly caused macroscopic cell death–like SARM1-TIR (Fig. 3A). This was unexpected, so we further examined whether disruption of the ThsA SLOG-motif enhanced background NADase activities (fig. S4). We generated three additional SLOG mutations (E403A, K388A, and K388E), and all had enhanced NADase activity relative to WT ThsA and did not require stimulation by ThsB-Auto (fig. S4, B and C). Regardless, these SLOG mutants illustrate that enhancing ThsA NADase activities can sharply deplete cellular NAD^+^ and trigger macroscopic cell death. Further, while the SLOG motif facilitates cADPR isomer binding and NADase activation, it also apparently regulates ThsA activation in the absence of signal. Collectively, these findings demonstrate that neither ThsB-Auto nor unstimulated WT ThsA depletes cellular NAD^+^ to an extent that is cytotoxic in planta.

We then validated that ThsA stimulation by ThsB-Auto required the TIR domain likely catalytic glutamate (E) residue (Fig. 3, C, D, and E). As in Fig. 2, we coexpressed ThsA WT with ThsB WT, ThsB-Auto, or ThsB-Auto E85Q and monitored for chlorotic cell death in *Nb eds1*^−/−^ plants (Fig. 3C). ThsA coexpression with ThsB-Auto elicited chlorosis, while coexpression with either ThsB WT or ThsB-Auto E85Q had no effect. Similarly, ThsB-Auto coexpression with NADase null ThsA N112A did not trigger cell death (Fig. 3C). ThsA and ThsB-Auto coexpression also sharply reduced cellular NAD^+^, as compared to control pairings of ThsB-Auto E85Q or ThsA N112A (Fig. 3D). To quantitatively assess cytotoxicity from ThsA stimulation by ThsB-Auto, we performed ion leakage assays on leaf discs (Fig. 3E). Briefly, during the onset of plant cell death, cellular ions leak into solution due to loss of membrane integrity. Consistent with the macroscopic cell death and NAD^+^ depletion observed in Fig. 3 (C and D), we recorded increases in ion conductivity when ThsA was coexpressed with ThsB-Auto, but not with ThsB-Auto E85Q or ThsA N112A loss-of-function controls (Fig. 3E). Together, these findings indicate that the catalytic activities of ThsB-Auto are required to stimulate ThsA-mediated NAD^+^ depletion and cell death. Furthermore, the functional inputs and outputs of the prokaryotic Thoeris system can be reconstructed in planta.

### A plant TIR, BdTIR, cross-activates ThsA, but ThsB-Auto does not cross-activate EDS1-mediated cell death

**Fig. 4.**
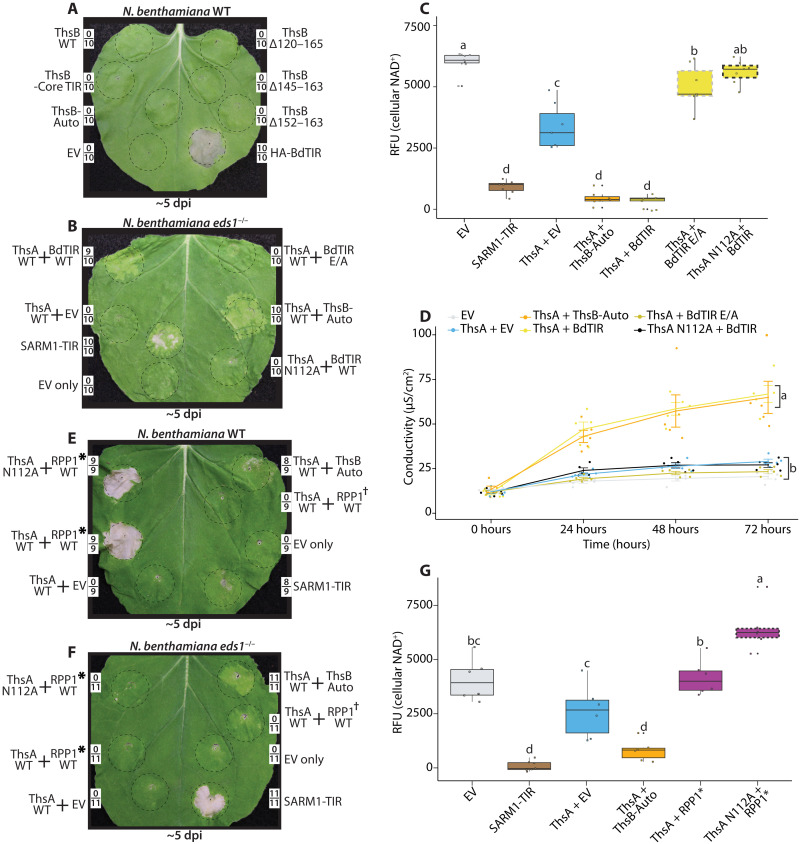
ThsB-Auto does not stimulate EDS1-mediated HR. BdTIR does stimulate ThsA, but the activated TIR-NLR, RPP1, does not. (**A**) *Nb* WT leaf ~5 dpi with constructs expressing HA-BdTIR, ThsB WT, ThsB-Auto, or the previously described ThsB C-terminal mutations. All constructs, including negative control EV, were expressed at an OD of 0.80. Framed numbers denote leaf replicates per set. (**B**) *Nb eds1*^−/−^ leaf ~5 dpi with constructs coexpressing ThsA or ThsA N112A with HA-BdTIR or BdTIR E/A. ThsA with ThsB-Auto and EV controls were also included. All coexpressions contained an OD of 0.40 of each construct, while SARM1-TIR and EV positive and negative controls were at an OD of 0.80. BdTIR E/A lacks TIR-domain catalytic residue. (**C**) Fluorescent NAD^+^ detection assay in *Nb eds1*^−/−^ leaves coexpressing ThsA, HA-BdTIR, or ThsB-Auto combinations, or positive and negative control SARM1-TIR and EV. NAD^+^ assays were performed 40 hpi. (**D**) Ion leakage assay in *Nb eds1*^−/−^ leaves coexpressing different ThsA and ThsB combinations. Leaf discs were collected ~72 hpi, and measurements were recorded every 24 hours for 3 days. Statistical analyses were performed for final time point. (**E** and **F**) *Nb* WT or *eds1*^−/−^ leaves ~5 dpi with constructs coexpressing ThsA, ThsB-Auto, or RPP1 TIR-NLR with cognate ATR1 effector. Asterisk denotes RPP1_WsB allele with activating ATR1-Emoy effector, while dagger has nonactivating ATR1-Emwa. ThsA was coexpressed at an OD of 0.40, and RPP1/ATR1 was expressed at an OD of 0.20 each. (**G**) Fluorescent NAD^+^ detection assay in *Nb eds1*^−/−^ leaves coexpressing noted ThsA, RPP1/ATR1, or ThsB-Auto combinations, as well as positive and negative control SARM1-TIR and EV. NAD^+^ assays performed 40 hpi. Similar experiments were performed at least three times. Statistical analyses: One-way ANOVA and Turkey HSD. Overlapping letters are ns difference (*P* > 0.05), while separate letter class indicates *P* < 0.05 or better.

The plant TIR BdTIR has been reported to activate ThsA, hinting that TIR signals from plant and bacterial immune systems might be conserved (*16*). After validating ThsB-Auto stimulation of ThsA in planta, we tested the reverse hypothesis: Does ThsB-Auto stimulate HR outputs by the plant TIR mediator, EDS1? Accordingly, we expressed BdTIR and ThsB-Auto in WT *Nb* and monitored HR (Fig. 4A). As in Fig. 1C, BdTIR signaled EDS1-mediated cell death; however, no cell death phenotypes were observed from ThsB-Auto, ThsB WT, or any ThsB variant (Fig. 4A). The finding that ThsB-Auto stimulates ThsA but is insufficient to activate EDS1 indicates that the signaling requirements for plant and Thoeris pathways are different. It further suggests that the enzymatic products of ThsB-Auto and plant TIRs are likely distinct.

Given the above lack of EDS1 activation by ThsB-Auto, we verified that BdTIR cross-activates ThsA (Fig. 4, B to D) (*16*). As in Fig. 3, we coexpressed ThsA with BdTIR or ThsB-Auto and examined NAD^+^ depletion and cell death (Fig. 4, B, C, and D). Similar to ThsB-Auto, BdTIR stimulated ThsA-mediated cytotoxicity and NAD^+^ consumption (Fig. 4, B, C, and D), confirming that signals from a plant TIR can also cross-active the Thoeris system in planta (*16*).

### The dicot TIR resistance protein, RPP1, signals EDS1-HR but does not stimulate ThsA

Although BdTIR activates EDS1-mediated HR in the dicot plant, *Nb*, *BdTIR* has no demonstrated immune functions within its source organism, the monocot plant *Brachypodium distachyon*. Therefore, we tested whether the TIR-NLR, RPP1, from the dicot *Arabidopsis thaliana*, also stimulated ThsA (Fig. 4, E and F). Accordingly, we expressed ThsA with ThsB-Auto or RPP1 and cognate ATR1 (*A. thaliana* recognized 1) effector. While ATR1 activated RPP1 to signal HR, ATR1 activation of RPP1 was unsuccessful at stimulating ThsA-mediated chlorosis or NAD^+^ depletion (Fig. 4, E and F). Subsequently, we examined in planta cADPR isomer production by effector-activated RPP1, relative to RPP1 E/A and BdTIR, using LC-MS (fig. S5). Similar to previous reports, BdTIR induced cADPR isomer accumulation to orders of magnitude greater than background levels in *Nb eds1^−/−^* plants, while activated RPP1 did not induce appreciable cADPR isomer accumulation (fig. S5) (*12*). The inability of ThsB-Auto to initiate EDS1-dependent cell death, along with the finding that activated RPP1 does activate EDS1, but not ThsA, further suggests that the TIR-produced immune signals for plant and prokaryotic TIR-systems are not conserved.

### ThsB-Auto and BdTIR produce different primary isomers of cADPR; ThsA is efficiently stimulated by 3′cADPR-producing TIRs

**Fig. 5.**
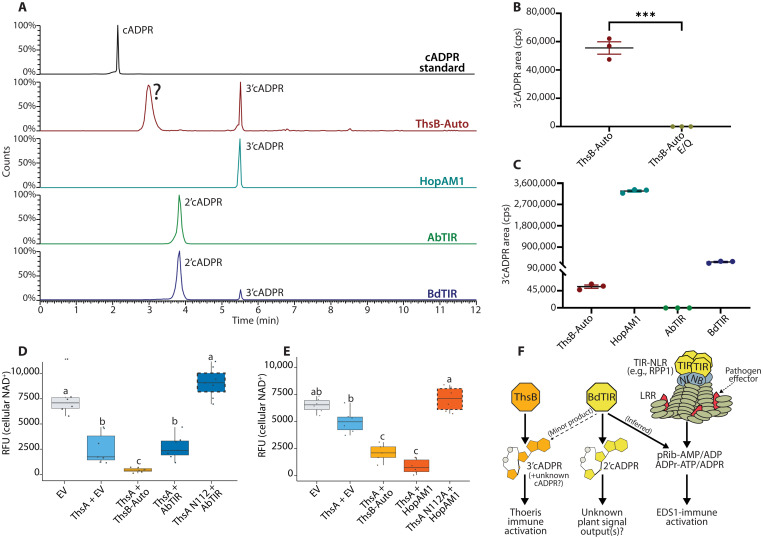
ThsB-Auto and BdTIR produce different cADPR isomers as major products; ThsA is highly stimulated by 3’cADPR. (**A**) LC-MS traces of cADPR isomers from in vitro NADase reactions of ThsB-Auto, HopAM1, AbTIR, or BdTIR, relative to cADPR standard. (**B** and **C**) In vitro production of 3’cADPR by ThsB-Auto relative to ThsB-Auto E85Q, HopAM1, AbTIR, or BdTIR. (***P < 0.005, *t*-test) (**D** and **E**) Fluorescent NAD^+^ detection assay in *Nb eds1*^−/−^ as previously described, except with AbTIR or HopAM1 coexpressed to stimulate ThsA. NAD^+^ assays were performed at ~40 hpi. Similar experiments were performed at least three times. (**F**) Model of Thoeris and EDS1-pathway stimulation by signals from plant and prokaryotic TIRs. Statistical analyses: One-way ANOVA and Turkey HSD. Overlapping letters are ns difference (*P* > 0.05), while separate letter class indicates *P* < 0.05 or better.

Recently, Eastman and colleagues (*14*, *20*) reported that the TIR effector protein, HopAM1, produced a cADPR-variant unlike that made by AbTIR; these two variant cADPRs have been identified as 2′cADPR (AbTIR product) and 3′cADPR (HopAM1 product). To determine which cADPR isomer(s) ThsB-Auto produced, we compared the in vitro NADase products of ThsB-Auto, BdTIR, AbTIR, and HopAM1 using LC-MS (Fig. 5A). The major cADPR isomer produced by ThsB-Auto had a unique retention time, unlike canonical cADPR standard or any products from BdTIR, HopAM1, or AbTIR (Fig. 5A). ThsB-Auto also produced a second peak, aligning with 3′cADPR made by HopAM1 (Fig. 5A). Unexpectedly, BdTIR generated both 2′cADPR (major product) and 3′cADPR (minor product), while AbTIR only produced 2′cADPR (Fig. 5A). Quantification of 3’cADPR peaks is shown in Fig. 5 (B and C). These findings provide a testable prediction: ThsA is stimulated by TIRs that produce 3′cADPR, but not 2′cADPR.

Ofir *et al.* (*16*) showed that ThsA was stimulated by cADPR-isomers from ThsB and BdTIR, but not canonical cADPR, while Manik *et al.* (*14*) found that ThsA orthologs from *Enterococcus faecium* (*Ef*ThsA) and *Streptococcus equi* (*Se*ThsA) were highly stimulated by 3′cADPR, but not 2′cADPR. Given that both BdTIR and ThsB-Auto make 3′cADPR and stimulate ThsA, we predicted that HopAM1 (3′cADPR producer) would stimulate ThsA, while AbTIR (2′cADPR) would not. We therefore coexpressed ThsA with AbTIR, or HopAM1, and assayed cell death and NAD^+^ depletion as before in *Nb eds1^−/−^* plants (Fig. 5, D and E, and figs. S6 and S7). AbTIR coexpression did not enhance ThsA-mediated cytotoxicity or NAD^+^ depletion relative to ThsB-Auto (Fig. 5D and fig. S6). Because HopAM1 is cytotoxic to *Nb* when highly expressed, we first titrated the HopAM1 delivery and found that an optical density at 600 nm (OD_600_) < 0.010 did not deplete NAD^+^ or trigger cell death (fig. S7). When coexpressed at an OD of 0.005, HopAM1 stimulated NAD^+^ depletion by ThsA similar to that promoted by ThsB-Auto (Fig. 5E).

ThsB-Auto stimulates ThsA and makes 3′cADPR, as well as an undefined cADPR isomer (Fig. 5A). However, whether other ThsB alleles might also produce this uncharacterized isomer was unclear. Therefore, we examined whether any of the previously screened ThsB orthologs (see fig. S2) could stimulate ThsA and whether they also produced this atypical isomer (fig. S8). The TIR domain of a second ThsB (ThsB2-TIR) encoded by *B. cereus* (MSX D-12) signaled ThsA-mediated cell death in *Nb*, while no other tested ortholog did (fig. S8) (*43*). Accordingly, ThsB2-TIR promoted ThsA-mediated NAD^+^ depletion, while in vitro LC-MS analysis revealed that ThsB2 produced 3’cADPR, but not the undefined isomer made by ThsB-Auto (fig. S8). Similar LC-MS analyses of *Nb eds1^−/−^* leaves expressing ThsB2, ThsB-Auto, and HopAM1 indicate that these generate cADPR isomers, which match the migration of 3′cADPR standard, while AbTIR-produced cADPR isomer migrates like 2′cADPR (fig. S9).

ThsB2-TIR more effectively stimulated ThsA-mediated cell death than the full-length ThsB2 protein, indicating that the N terminus may affect NADase activities (fig. S9). ThsB (192 amino acids) and ThsB2 (193 amino acids) differ in length by just one residue; however, their overall amino acid identity is low (<20%). AlphaFold predictions suggest that ThsB and ThsB2 do not share structural homologies outside the core-TIR domain (fig. S8). Notably, ThsB2-TIR did not require BB-loop modifications to stimulate ThsA, suggesting that the NADase activities of ThsB and ThsB2 could be regulated differently.

These findings identify 3′cADPR as an efficient trigger for ThsA and suggest that ThsB-Auto–produced 3′cADPR is the activating signal for the Thoeris system. We further explain the cross-stimulation of the Thoeris system by BdTIR: BdTIR generates multiple isomers of cADPR, including the 3′cADPR made by ThsB-Auto. The model in Fig. 5F summarizes these results, while fig. S10 lists the known enzymatic products of each TIR and their EDS1/ThsA stimulation phenotypes.

## DISCUSSION

Plant and prokaryotic TIRs use enzymatic activities to produce immune signals in response to pathogen challenges (*3*, *4*, *15*, *16*). Deciphering the unique structures and physiological outputs of TIR signals is progressing but remains challenging (*3*, *9*–*12*, *40*, *41*). In this report, we generated an autoactive variant of the prokaryotic TIR, ThsB, and reconstructed Thoeris inputs and outputs in planta, to test the hypothesis that plant and prokaryotic signals are conserved. We found that the signals required to activate EDS1 and ThsA were not conserved, and we identify ThsB-generated 3′cADPR as an activator of the Thoeris mediator, ThsA. ThsB-Auto also produces an undefined cADPR isomer, which remains a potential ThsA activator. We demonstrate that some plant TIRs simultaneously produce both 2′cADPR and 3′cADPR and provide a plausible explanation for the cross-kingdom activation of the Thoeris system by the plant BdTIR. Plant TIRs can generate numerous types of putative signals and even use nucleotide substrates beyond NAD^+^ (*9*–*11*, *14*).

The detection of bacteriophage triggers ThsB NADase activities, although how ThsB mechanistically transitions to an active state is not understood (*16*). Plant TIR-NLRs are also activated by pathogen detection, and cryo-EM studies indicate that a conserved BB-loop shifts during activation to influence substrate access and TIR-TIR associations (*27*, *28*). This conserved TIR loop is also critical for the catalytic activity of the prokaryotic virulence factor, AbTir (*14*). Modifying this analogous loop within ThsB generates an autoactive variant; however, it is unknown if phage detection effects similar conformational changes within ThsB during activation. Among the plant TIR domains tested, similar BB-loop alterations had the opposite phenotype, resulting in a loss of function. This indicates that plant and prokaryotic Thoeris TIRs may have distinct requirements for catalysis.

During the preparation of this manuscript, two independent studies on the Thoeris system were published by Leavitt *et al.* (*41*) and Manik *et al.* (*14*). Similar to Manik *et al.* (*14*) and the report here, Leavitt *et al.* (*41*) identified 3′cADPR as the Thoeris activation signal. Leavitt *et al.* (*41*) also identified a phage-encoded repressor of Thoeris, Tad1 (Thoeris antidefense 1), which blocks ThsA activation by sequestering cADPR isomers. Although a Tad1 structure with bound 2′cADPR was generated, this cADPR signal was derived from BdTIR and not from ThsB. Critically, Tad1’s specificity for different cADPR isomers is not exclusive, and Tad1 can sequester both 3′cADPR and 2′cADPR (*41*). Thus, our finding that 3′cADPR-producing TIRs (ThsB-Auto, ThsB2, HopAM1, and BdTIR) effectively signal ThsA and that 2′cADPR is the major NADase product of BdTIR agrees with both reports (*14*, *41*). We find that BdTIR also generates 3′cADPR in minor amounts, which provides an explanation for the cross-activation of ThsA reported by Ofir *et al.* (*16*). In the report of Manik *et al.* (*14*), ThsB products were not directly examined, but rather, purified isomers of 2′ and 3′cADPR were assayed for ThsA stimulation. Consistent with our studies, an *Ef*ThsA was found to be ~100-fold more sensitive to 3′cADPR (*14*). It remains possible that ThsA might be further stimulated by the second, and structurally unresolved, cADPR isomer that we detected from ThsB-Auto in vitro.

Similar to previous in vitro reports, BdTIR cross-activated ThsA in our in planta assays, indicating some conservation of produced metabolites among plants and prokaryotic Thoeris (*16*). However, in the reciprocal experiment, ThsB-Auto did not cross-activate plant EDS1-dependent TIR pathways. Recent reports find that the plant TIR–produced metabolites pRib-AMP/ADP and ADPr-ATP/ADPr promote EDS1 family heterodimer formation and association with the downstream helper NLRs, ADR1, and NRG1 (*9*, *10*). While it has not been formally demonstrated that 2′cADPR is dispensable for EDS1 pathway activation in vivo, pRib-AMP/ADP or ADPr-ATP/ADPr appears sufficient in vitro to link the EDS1 complex to downstream helper NLR oligomerization (*9*, *10*). v-cADPR (2′cADPR) was initially described as a biomarker of plant TIR activity (*12*, *13*). However, this study, similar to that of Duxbury *et al.* (*40*), provides further evidence that v-cADPR (2′cADPR) alone does not activate EDS1-dependent immune signaling. Rather, the cell death observed from sustained overexpression of AbTIR and TcpO-TIR is linked to cellular NAD^+^ depletion and is similar to the EDS1-independent death caused by SARM1-TIR (*12*). Despite this, it remains possible that 2′cADPR and 3′cADPR could have alternate (EDS1-independent) functions in plant immunity or could act as signals in presently unknown pathways. Alternatively, in planta 2′cADPR and/or 3′cADPR produced by TIR proteins could be immunologically irrelevant side products. Thus, it is still unclear why plant TIR–only proteins produce v-cADPR (2′cADPR) and why TIR-only proteins such as BdTIR generate ~100-fold more in planta relative to TIR domains of examined TIR-NLR proteins (*12*).

Similar to ThsB-Auto and HopAM1, BdTIR also generates 3′cADPR, although to a much lesser extent than 2′cADPR. Given that 3′cADPR is produced by the phytopathogen effector HopAM1, if it is a signaling molecule, it presumably might act as a negative regulator of plant immunity. Curiously, plant TIR–only proteins also generate 2′,3′cNMPs, which can regulate the formation of stress granules (*11*, *44*). Future studies will examine how TIR-generated 3′cADPR or 2′,3′cNMP affect stress or associated transcriptional defense responses and if elevated levels of 2’cADPR could have signaling roles independent of EDS1.

In addition to 3′cADPR, we detected an unknown isomer of cADPR generated by ThsB-Auto. The discovery of Tad1 indicates that phages are evolving to circumvent the Thoeris system (*41*). Accordingly, Thoeris systems are under selective pressure to overcome Tad1 inhibition. Certain Thoeris operons encode several ThsB paralogs, although it is not clear if ThsB stacking might further benefit immunity beyond potentially enabling the detection of multiple phage types (*43*). For instance, an expanded ThsB arsenal could enable enzymatic outputs to diversify and produce different signal molecule types and/or have altered kinetics. ThsA orthologs have varying sensitivity to different TIR-derived products (*14*). ThsA activation by particular cADPR isomers might also be under selection in response to Tad1-containing phages (*41*). It will be interesting to assess ThsA stimulation by the unknown cADPR isomer produced by ThsB-Auto and if Tad1 similarly blocks this putative signal as well.

We do not yet know the mechanism by which BB-loop modification enables ThsB autoactivity. Similar substation of glycine residues within the BB-loop of a prokaryotic TIR–STING (stimulator of interferon genes) protein results in a loss of NADase functions (*45*). However, TIR-STING proteins lack apparent cyclase activity, suggesting differences in the catalytic mechanism, as compared to ThsB TIRs (*45*). ThsB2-TIR did not require BB-loop modifications to stimulate ThsA, suggesting that ThsB and ThsB2 NADase activities may be differentially regulated. While BB-loop alteration confers ThsB autoactivity, it is possible that this change could shunt NADase outputs from 3′cADPR generation toward production of the unknown cADPR isomer. The finding that HopAM1 and ThsB2 can both stimulate ThsA but do not produce the structurally undefined isomer made by ThsB-Auto indicates that this isomer is at least not required for ThsA activation. Regardless, these findings indicate that the targeted alteration of TIR BB-loops might allow for the generation of distinct and potentially useful products by TIRs.

Unstimulated ThsA has detectable NADase activities in vitro (*14*, *16*, *33*). While our in planta reconstruction showed that ThsA by itself does diminish NAD^+^ levels relative to the catalytically inactive SIR2 mutant (N112A), cytotoxic phenotypes were only apparent after ThsA stimulation by ThsB-Auto. Manik *et al.* (*14*) assayed other ThsA orthologs and found that *Ef*ThsA and *Se*ThsA had lower unstimulated NADase activities in vitro relative to the prototypic ThsA of *B. cereus* MSX-D12. It is possible that this unstimulated “background” NADase activity of ThsA in vitro is influenced by recombinant protein preparation. Whether Thoeris systems impose host fitness penalties is not clear (*46*); however, because ThsA can be readily expressed in *Escherichia coli*, it presumably is not strongly cytotoxic to host cells (*14*, *16*, *33*, *41*). Further studies could examine whether the SLOG domain, in the absence of ThsB signals, is influenced by other cellular metabolites or regulators that act to restrain unstimulated ThsA NADase activities.

We generated ThsA R371A, which reportedly prevents stimulation by ThsB-generated signals (*14*, *16*). However, R371A, as well as SLOG substitutions at E403 or K388, resulted in a constitutive enhancement of NADase activity that was cytotoxic in planta. A recent crystal structure of the ThsA SLOG domain shows that these three residues coordinate 3′cADPR binding and subsequently influence NAD^+^ access to the catalytic SIR2 domain (*14*). Within *Ef*ThsA, substituting these residues resulted in a loss of sensitivity to 3′cADPR, at least in vitro (*14*). Why mutating these residues within ThsA (*B. cereus* MSX D-12) promotes autoactivity in planta is unclear, although it is possible that these substitutions could enhance ThsA sensitivity to other metabolites within plant cells (*14*). Regardless, we provide evidence that the SLOG domain likely has roles in both inhibition and activation. Whether SLOG domains from phylogenetically distant ThsA proteins are equally stimulated by 3′cADPR versus other TIR-derived metabolites is not known. Analyzing the conservation of SLOG residues among ThsA orthologs may provide clues into whether other Thoeris systems might use unique ThsB-generated signals.

Enzymatic TIRs are prevalent in the immune systems of prokaryotes and eukaryotes, and their roles are highly adaptable. For instance, Thoeris TIRs generate immune signals, while other TIRs act as executioners that simply deplete NAD^+^ (*43*, *45*, *47*–*49*). NLR-like immune proteins have been reported in prokaryotes, and some unicellular algae encode plant-like TIR-NB-LRR proteins (*19*, *50*, *51*). Evolution adapts existing genetic modules into diverse roles, and whether plant TIR pathways can be directly traced to particular bacterial or unicellular eukaryote lineages remains an open question (*52*). Further study of the TIR domains encoded by prokaryotes and early plant lineages will shed light on the conservation of TIR signals and likely reveal additional types of TIR-generated immune signals.

## MATERIALS AND METHODS

### Immunoblots

Three 6-mm discs from three different transformed *Nb* leaves were harvested into a 2.0-ml tube containing a single glass bead and flash-frozen in liquid nitrogen. Tissue was homogenized in a TissueLyzer II (QIAGEN) at 30 Hz for 30 s. Proteins were extracted in 200 μl of lysis buffer [50 mM tris-HCl (pH 7.5), 150 mM NaCl, 5 mM EDTA, 0.2% Triton X-100, 10% (v/v) glycerol, and 1/100 Sigma-Aldrich protease inhibitor cocktail]. Lysates were centrifuged at 4°C for 5 min at 5000 rpm and stored on ice. Equal volumes of lysates were loaded in each sample lane for analysis by SDS–polyacrylamide gel electrophoresis (PAGE). After transfer, nitrocellulose blots were blocked for 1 hour at room temperature in 3% (w/v) nonfat dry milk TBS-T (50 mM tris, 150 mM NaCl, and 0.05% Tween 20). Immunoblots for either HA (3F10, Roche) or green fluorescent protein (GFP; catalog no. 1181446001, Roche) were incubated for 1 hour at room temperature in 3% (w/v) nonfat dry milk TBS-T at 1:2000, followed by 3× washes in TBS-T. Secondary horseradish peroxidase–conjugated goat anti-rabbit or goat anti-mouse immunoglobulin G (MiliporeSigma) was added at 1:10,000 in TBS-T (3% milk) and incubated for 1 hour at room temperature on a platform shaker, followed by 3× washes with TBS-T. Chemiluminescence detection was performed with Clarity Western ECL Substrate (Bio-Rad) and developed using a ChemiDoc MP chemiluminescent imager (Bio-Rad).

### Transient agrobacterium expression in *Nb*

*Agrobacterium tumefaciens* strain GV3101 was syringe-infiltrated at an OD_600_ of 0.80 into young leaves of ∼4- to 5-week-old *Nb* plants. Viral suppressor of silencing p19 was included within *Nb* infiltrations at an OD of 0.05, as described previously (*12*, *35*). GV3101 liquid cultures were grown overnight at 28°C in rifampicin (50 μg/ml), gentamicin (50 μg/ml), and spectinomycin (50 μg/ml). Cultures were induced ∼3 hours in 10 mM MES buffer (pH 5.60), 10 mM MgCl_2_, and 100 μM acetosyringone before infiltration. *Nb* plants were grown in a Percival set at 25°C with a photoperiod of 16-hour light at 80 μE/m^2^ per second. For Thoeris system reconstruction assays, GV3101 cultures at an OD of 0.80 were mixed 1:1 with respective constructs before coinfiltration, unless otherwise noted in figure panel.

### Protein structure modeling

Protein structure homology models for full-length AbTir and TcpO proteins were generated using AlphaFold (*53*), and resulting Protein Data Bank (PDB) files were analyzed with PyMOL (The PyMOL Molecular Graphics System, Version 1.8 Schrödinger LLC). ThsB (PDB: 6LHY) and ThsA (PDB: 6LHX) (*B. cereus* MSX D-12) structures were previously generated in (*33*). RPP1 (PDB: 7CRC) cryo-EM structure was generated in (*28*).

### DNA vector construction and polymerase chain reaction mutagenesis

DNA fragments encoding codon-optimized ThsB and ThsA (from *B. cereus* MSX D-12) and other ThsB-orthologs were synthesized by Twist Biosciences with BP-compatible recombination ends. All DNA fragments were BP-cloned into vector pDONR207 using Gateway BP Clonase (Invitrogen) and sequence-verified (GeneWiz). Open reading frames were cloned using Gateway LR Clonase (Invitrogen) into previously described binary vectors containing Omega leader sequences: pGWB602 (35S Omega:), pGWB615 (35S Omega: HA-), pGWB641 (35S Omega: -GFP), and pGWB602 (35S: HA_SAM, Sterile alpha motif domain from SARM1-). The polymerase incomplete primer extension method was used to introduce single amino acid substitutions or small insertions/deletions (*54*). Polymerase chain reaction was performed on genes within pDONR207 constructs with Q5 High-Fidelity polymerase (New England Biolabs, Ipswich, MA), and all constructs were sequence-verified by GeneWiz before cloning into binary vectors. The HA_SAM oligomerization domain (1× HA tag–SAM_478–578_-GGGGS) of SARM1, the SARM1-TIR domain (residues 561 to 724), and the TcpO-TIR domain (residues 204 to 341) were described previously in (*12*). The AbTIR corresponds to residues 157 to 292. Truncated ThsB2 is residues 44 to 193. Catalytic glutamate (E) residues are as follows: AbTir (E208), TcpO-TIR (E279), BdTIR (E127), ThsB (E85), and ThsB2 (E124). Amino acid sequences for TIR and ThsA constructs used are provided in the Supplementary Materials.

### Recombinant protein expression and purification

Corresponding ThsB, AbTIR, HopAM1, and BdTIR constructs were cloned into the pET30a^+^ vector with N-terminal Strep II tag and C-terminal 6× His tag. Proteins were expressed in *E. coli* BL21 (DE3) and induced overnight using the autoinduction method (*55*). Cell pellets were resuspended in lysis buffer [50 mM Hepes (pH 8.0), 300 mM NaCl, and 1 mM dithiothreitol (DTT)] and lysed using sonication. The resulting supernatant was filtered and applied onto NiA beads. The beads were washed with lysis buffer. Bound proteins were eluted using elution buffer [50 mM Hepes (pH 8.0), 300 mM NaCl, and 250 mM imidazole]. Eluted proteins were concentrated and applied onto a Superdex 75 HiLoad 26/60 size exclusion column (GE Healthcare) preequilibrated with gel filtration buffer [10 mM Hepes (pH 7.5), 150 mM NaCl, and 1 mM DTT]. The peak fractions were pooled and confirmed by SDS-PAGE and concentrated using Amicon Ultra-15 Centrifugal Filter Units (Millipore). The protein samples were stored at −80°C.

### In vitro NADase assays

Ten microliters of NiA beads bound with purified proteins were incubated with 30 μM NAD^+^ (final concentration) in 50 μl of buffer (92.4 mM NaCl and 0.64× phosphate-buffered saline). Reactions were carried out at room temperature (around 25°C) for 2 hours, stopped by addition of 50 μl of 1 M perchloric acid (HClO_4_), and then placed on ice for 10 min. Neutralization was performed by adding 16.7 μl of 3 M K_2_CO_3_. Samples were placed on ice for another 10 min and then cleared by centrifugation. Extracted metabolites were stored at −20°C until later LC–tandem MS (MS/MS) analysis.

### In vitro LC-MS/MS metabolite measurements

The samples were centrifuged at 12,000*g* for 10 min, and the cleared supernatant was applied to the LC-MS/MS for metabolite identification and quantification. Samples were analyzed by Q Exactive quadrupole orbitrap high-resolution mass spectrometry coupled with a Dionex Ultimate 3000 RSLC (HPG) ultraperformance liquid chromatography (UPLC-Q-Orbitrap-HRMS) system (Thermo Fisher Scientific) and a heated electrospray ionization source under positive ion modes . The injection volume was set to 1 μl. Samples were separated with an ACQUITY UPLC HSS T3 column (100 mm by 2.1 mm, 1.8-μm particle size; Waters). The mobile phase consisted of 2 mM ammonium formate in water (A) and in 100% methanol (B), and the gradient elution was set as the following: 0 to 2.00 min, 1% B; 2.00 to 7.00 min, 1 to 95% B; 9.00 to 9.10 min, 95 to 1% B; 9.10 to 11.00 min, 1% B. The flow rate was set to 0.3 ml/min and column temperature at 45°C. Metabolites were quantified by using area, and the retention time for each compound was determined using standards including NAD^+^, cADPR, and Nicotinamide (Nam) dissolved in 50% methanol. The metabolites were also detected and quantified with a Triple Quad mass spectrometer (6500; Agilent) under positive ESI multiple reaction monitoring (MRM) for monitoring analyte parent ion and product ion formation. MRM conditions were optimized using authentic standard chemicals including the following: NAD^+^ [(M + H) + 664 > 136.00, 664 > 428, 664 > 542]; Nam [(M + H) + 123 > 80]; cADPR [(M + H) + 542 > 136, 542 > 348, 542 > 428]; v-cADPR [(M + H) + 542 > 136].

### In planta LC-MS analysis

LC-MS/MS analysis was carried out on Waters TQ-XS triple quad mass spectrometer coupled with a Waters Acquity H-class UPLC-C18 column (1.7 μm, 2.1 mm by 50 mm). Mobile phases were 2 mM ammonium acetate (A) and 100% methanol (B); flow rate is 0.2 ml/min; and gradients were 0 to 5 min, 0% B; 5 to 7 min, 0 to 20% B; 7 to 8 min, 20 to 100% B. Then, the column was washed with 100% B for 2 min before equilibration to 100% A over 15 min. Mass spectrometer conditions are as follows: capillary voltage of 800 V, desolvation temperature at 600°C, desolvation gas (nitrogen, 1000 liters/hour), cone gas of 150 liters/hour, and nebulizer gas of 7 bar. MRM parameters for the detection of cADPR isomers are 542/136 (cone voltage of 20 V and collision energy of 32 eV) and 542/348 (cone voltage of 20 V and collision energy of 28 eV). cADPR isomers were verified by comparing with authentic standards, including 2′cADPR and 3′cADPR.

### In planta NADase assays

Three 6-mm discs of transformed *Nb* leaf tissue were harvested into 2.0-ml tubes containing a single glass bead and flash-frozen in liquid nitrogen. Tissue was homogenized in a QIAGEN TissueLyzer II at 30 Hz for 30 s. Tissue was resuspended in 300 μl of ice-cold lysis buffer [50 mM tris-HCl (pH 7.5), 150 mM NaCl, 5 mM EDTA, 0.2% Triton X-100, and 10% (v/v) glycerol] and stored on ice. Lysates were centrifuged at 4°C for 5 min at 5000 rpm and returned on ice. Supernatant was diluted 1:3 in lysis buffer before addition to the Amplite NAD^+^ Assay Kit (catalog no. 15280, AAT Biosciences). Colorimetric reagent was developed for 20 min at room temperature. Colorimetric NAD^+^ detection was performed in 96-well black bottom plates (Costar) on an Infinite M Plex (Tecan) plate reader at excitation (420 nm) and emission (480 nm) using i-control 2.0 software and presented as relative fluorescence units.

### Ion leakage assays

Ion leakage assays were performed essentially as described in (*35*). Briefly, 6 mm–by–4 mm leaf discs were collected from *Nb* plants at ~3 dpi and placed into sterile 15-ml conical tubes containing 6.0 ml of ultrapure H_2_O. Ion measurements were recorded at noted times using an OrionStar A112 conductivity meter (Thermo Fisher Scientific). Statistical comparison performed for end time point (72 or 96 hours).

### Statistical analyses

Multiple comparisons were analyzed via one-way analysis of variance (ANOVA) with post hoc Tukey HSD (honestly significant difference) using R Studio [version 1.4.1717, RStudio Team (2021), Boston, MA]. Comparisons of significance indicated with compact letter display (CLD). Overlapping letters are nonsignificant (*P* > 0.05), while separate letter classes indicate *P* < 0.05 or better.

## Supplementary Materials

### This PDF file includes:

Figs. S1 to S10 Legends for datasets S1 and S2

## Notes

View/request a protocol for this paper from *Bio-protocol*.
