# Supplementary material for: Plant and prokaryotic TIR domains generate distinct cyclic ADPR NADase products
Source: Sci Adv. 2023 Mar 17;9(11):eade8487. doi: 10.1126/sciadv.ade8487 (PMC10022894; doi:10.1126/sciadv.ade8487)
Supplement: Supplementary file 1 — Figs. S1 to S10 Legends for datasets S1 and S2 [file sciadv.ade8487_sm.pdf]

Science Advances
AAAS

Supplementary Materials for

Plant and prokaryotic TIR domains generate distinct cyclic ADPR NADase products

Adam M. Bayless *et al.*

Corresponding author: Marc T. Nishimura, marc.nishimura@colostate.edu; Li Wan, lwan@cemps.ac.cn

Sci. Adv. **9**, eade8487 (2023)
DOI: 10.1126/sciadv.ade8487

The PDF file includes:

Figs. S1 to S10
Legends for datasets S1 and S2

Other Supplementary Material for this manuscript includes the following:

Datasets S1 and S2

SI 1

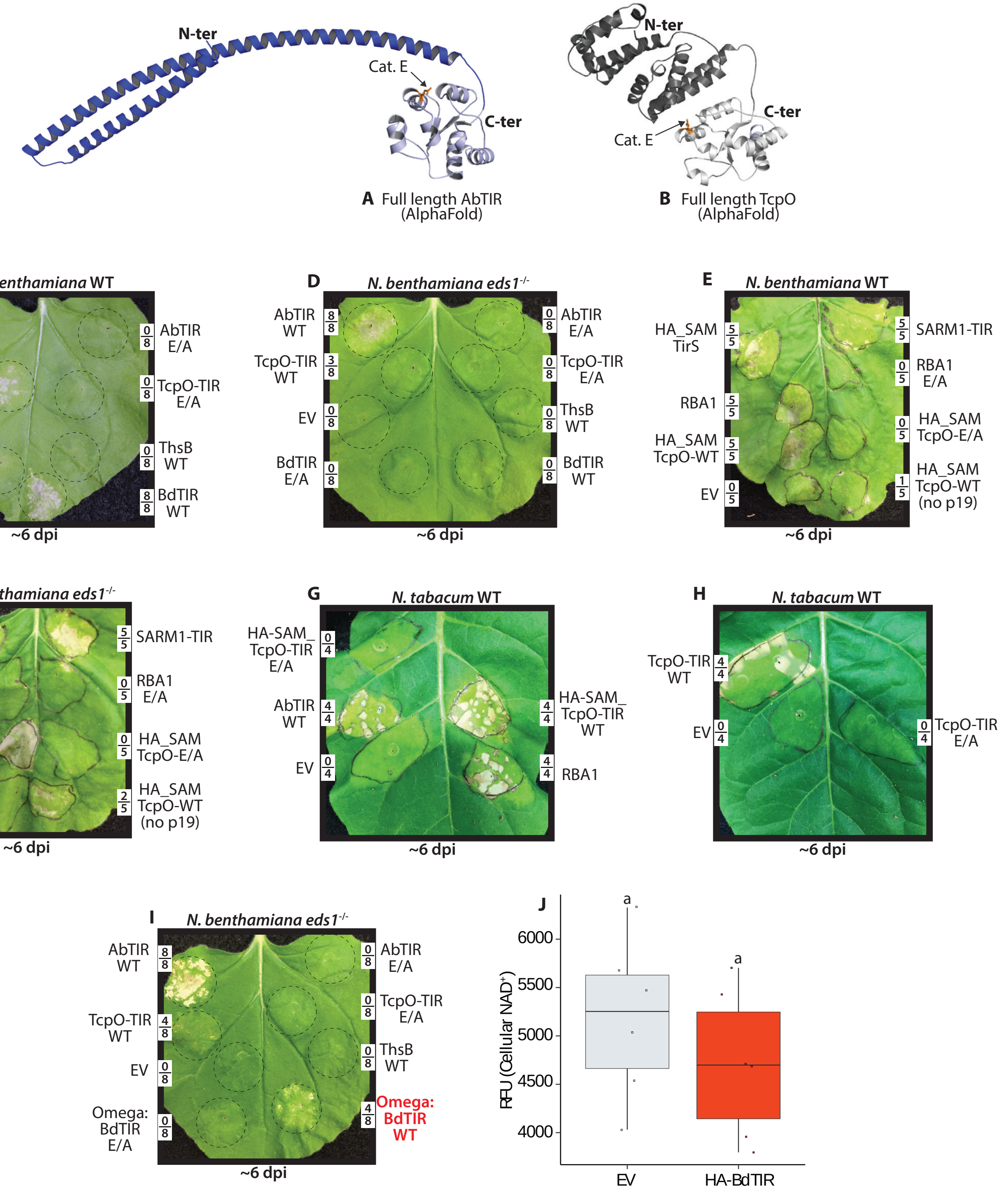

SI 1. Over-expression of prokaryotic TIRs can trigger EDS1-independent cell death. (*A* and *B*) AlphaFold models of full length AbTir encoded by human pathogen, *Acinetobacter baumanii*, or full length TcpO encoded by *Methanobrevibacter olleyae*. TIR-domain core is colored grey; catalytic glutamate residue (E) is colored orange. (*C-F*) *Nb* WT or *eds1*$^{-/-}$ leaves expressing untagged AbTIR or TcpO-TIR, or HA-SAM-tagged versions of AbTIR, TcpO-TIR or TirS. TirS is a previously described ADPR-producing TIR from *Staphylococcus aureus* (*26*). SAM (sterile alpha motif of SARM1) oligomerization domain fusions promote TIR-activity, as previously described (*12*). EV: 35S:GFP. Positive HR-control RBA1 (Response to HopBA1) previously described in Nishimura et al (*35*). All constructs infiltrated at OD 0.80 and imaged ~6 dpi. (*G-H*) Like C, but Agro-infiltration of TIRs into WT *Nicotiana tabacum*. (*I*) 35S Omega leader driven expression of BdTIR can also drive EDS1-independent cell death in *Nb*. All constructs infiltrated at OD 0.80 and imaged ~6 dpi. (*J*) Fluorescent NAD$^+$-detection assay in *Nb eds1*$^{-/-}$ leaves performed at 40 hpi. 35S binary constructs expressing 35S:HA-BdTIR or EV (empty vector, 35S: GFP). Similar experiments were performed at least three times. Statistical analyses: One-way ANOVA and Turkey HSD. Over-lapping letters are ns (non-significant) difference (p>.05) while separate letter class indicates p<.05 or better.

SI 2

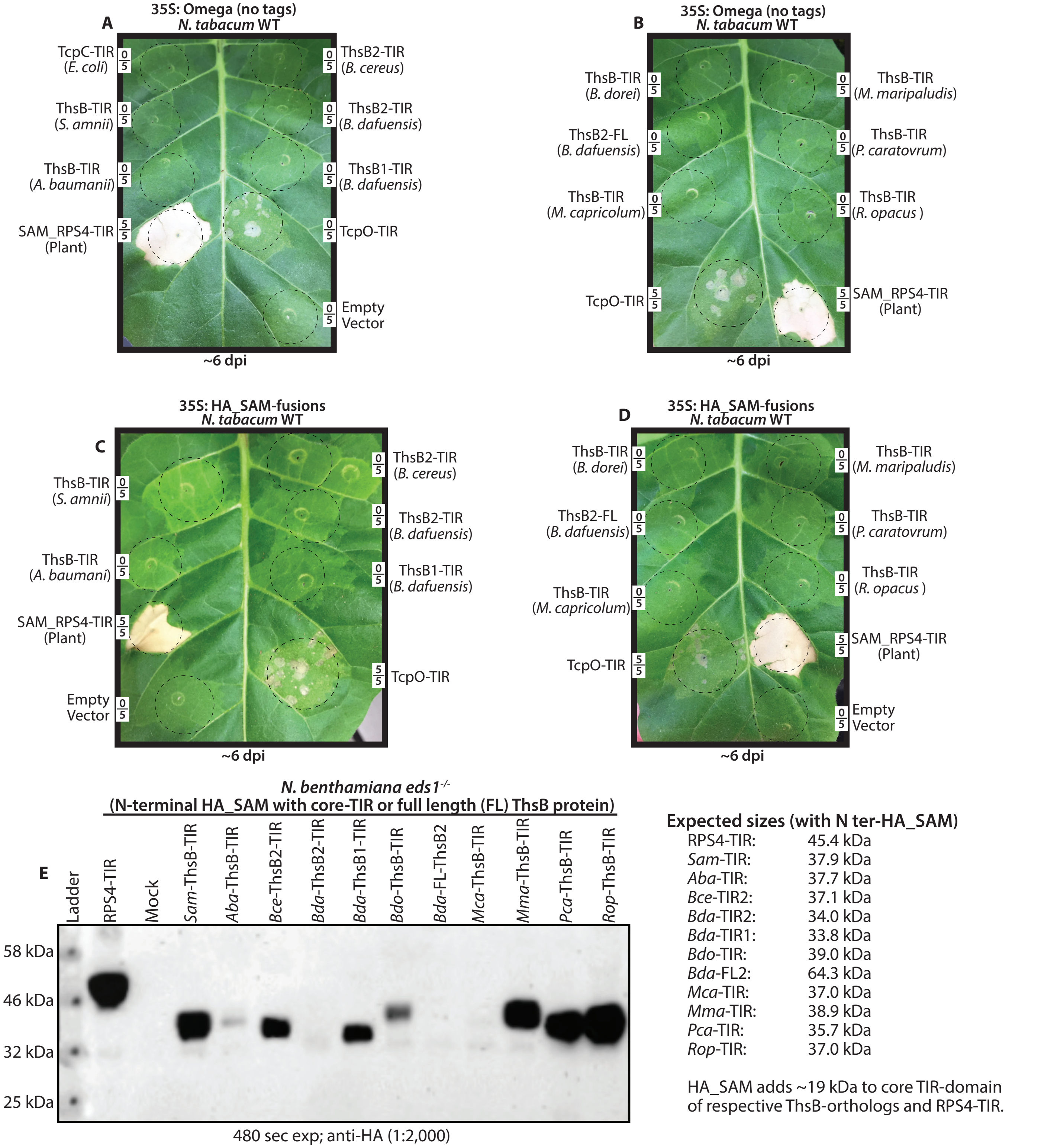

SI 2. Examined orthologs of ThsB do not trigger HR. (*A-D*) *Nicotiana tabacum* leaves expressing core TIR-domains (or full-length protein (FL)) of noted ThsB-orthologs, either with or without N-terminal SAM-oligomerization domain fusions. Leaves shown ~5-6 dpi; all constructs infiltrated at OD 0.80. Framed numbers denote leaf replicates per set. Similar experiments were performed at least three times. (*E*) Anti-HA immunoblot detection of HA-SAM_ThsB-orthologs harvested from *Nb eds1*$^{-/-}$ leaves at ~40 hpi. Expected size of HA_SAM-fusion proteins and origin of TIR-domain listed on right. Positive HR-control SAM_RPS4-TIR fusion previously described by Wan et al (*12*).

SI 3

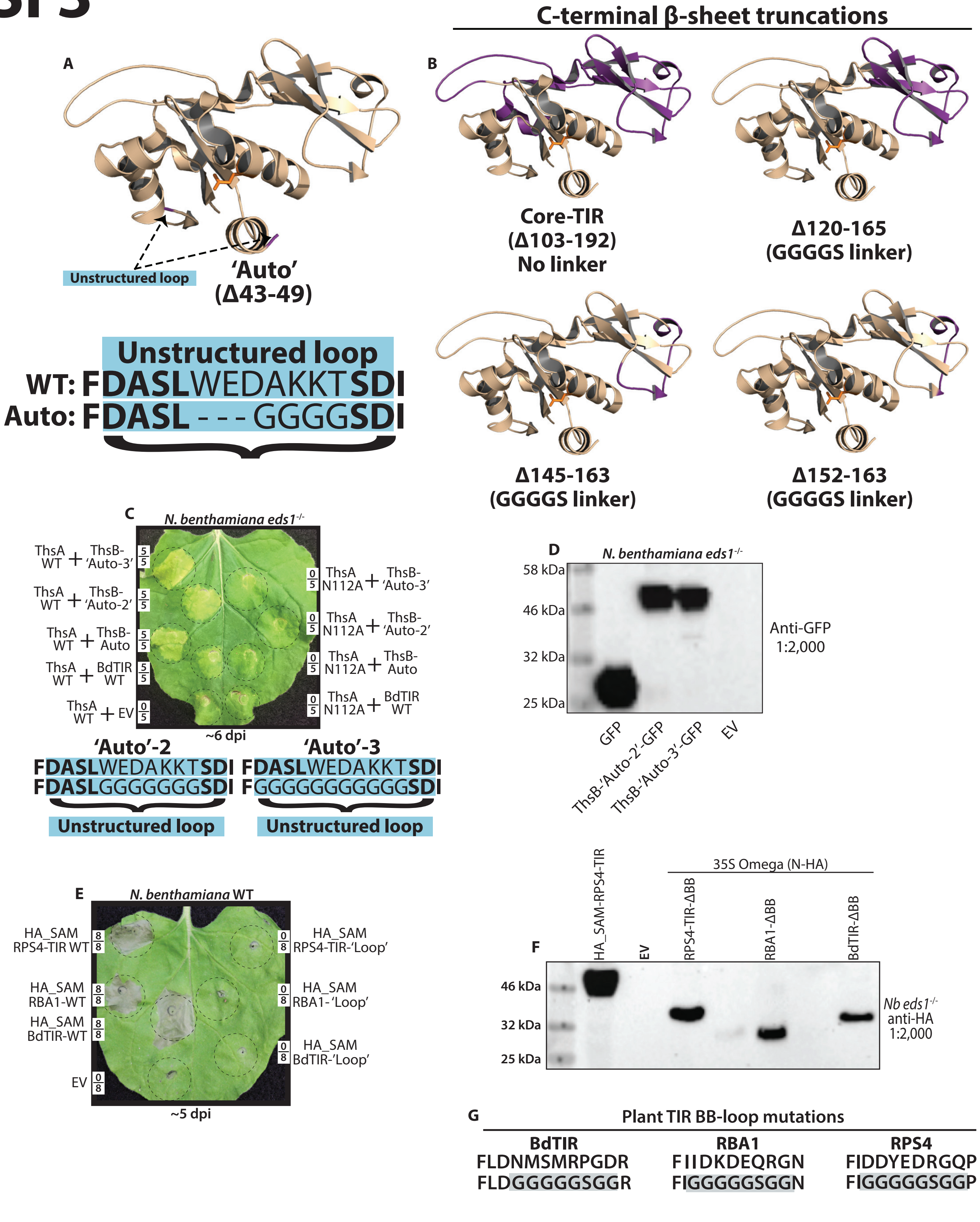

SI 3. BB-loop substitutions promote ThsB auto-activity but impair constitutively active plant TIRs. (*A*) Crystal structure of ThsB (PDB ID: 6LHY) determined by Ka et al (*33*), showing unstructured BB-loop region replaced in the ThsB-Auto active mutant (see arrows). Sequence of ThsB-Auto shown below. (*B*) Like A, but structures indicate ThsB C-terminal replacement variants. Replaced C-terminal regions are shown purple. (*C*) *Nb eds1*$^{-/-}$ leaf expressing additional auto-active mutants of ThsB. All constructs infiltrated at OD 0.80 and imaged ~6 dpi. Framed numbers denote leaf replicates per set. Sequences of mutants shown below. (*D*) Immunoblot of N-HA tagged versions of ThsB-Auto-2 and -3, respectively. (*E*) *Nb* WT leaf indicating phenotypes of plant TIR BB-loop replacement constructs, relative to WT versions. (*F*) Immunoblot of HA-tagged BB-loop replacements within plant TIRs. (*G*) Sequence of WT (Top) and BB-loop (bottom) replacements within BdTIR, RBA1 or RPS4, respectively.

SI4

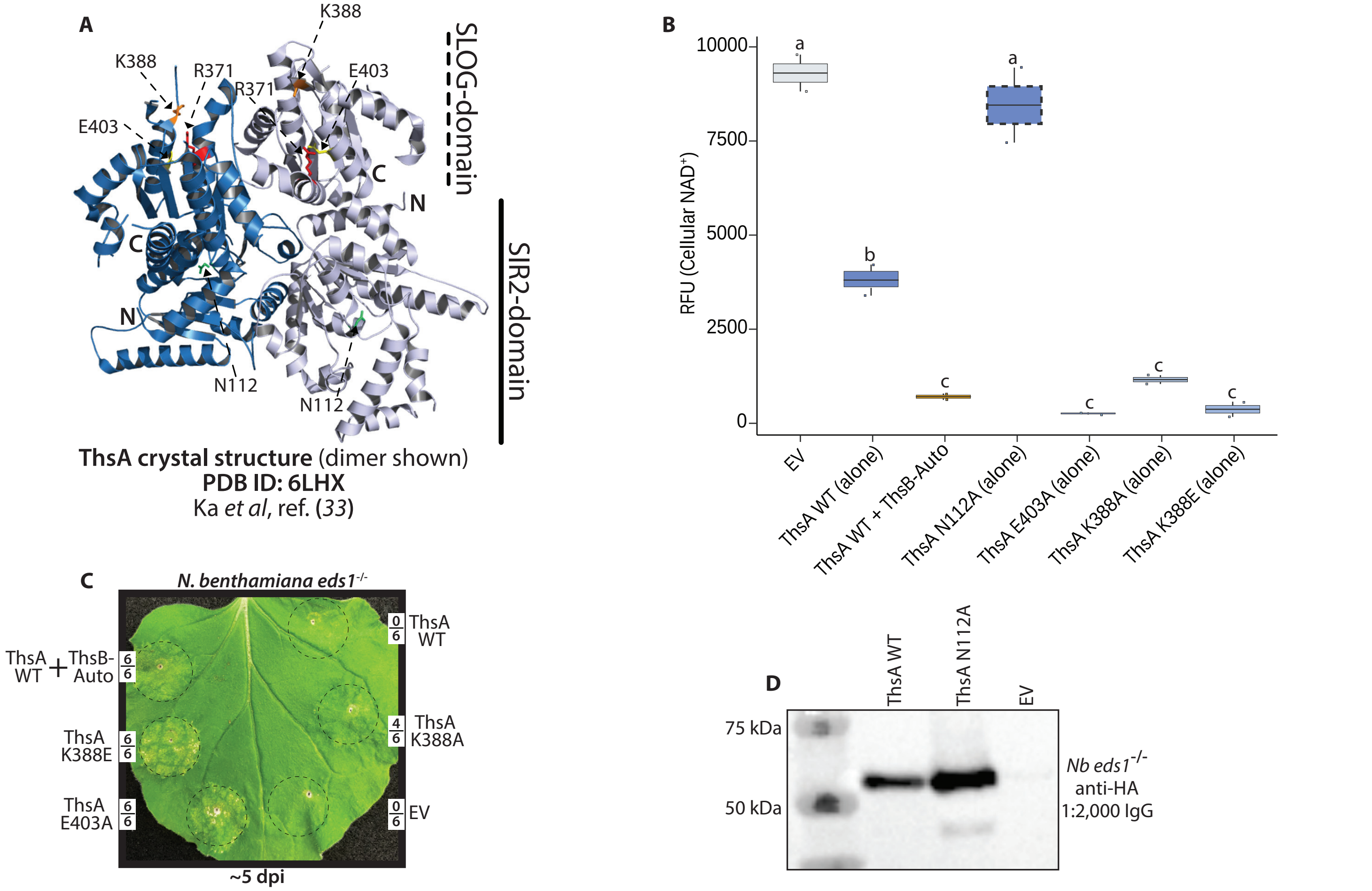

SI 4. Substitution of ThsA-SLOG domain residues causes NADase auto-activity and cytotoxicity in planta. (*A*) Crystal structure of ThsA (PDB ID: 6LHX) determined by Ka *et al*. (*33*); two ThsA monomers are shown in blue and grey. N: N-terminus, C: C-terminus. N112 of SIR2 domain shown green, while R371, K388, and E403 within the SLOG-domain are colored red, orange, or yellow, respectively. (*B*) Fluorescent NAD$^+$-detection assay in *Nb eds1$^{-/-}$* leaves expressing various ThsA constructs alone, or ThsA + ThsB-Auto. Individual constructs expressed at OD 0.80, and tissue harvested ~40 hpi. (*C*) *Nb eds1$^{-/-}$* leaf expressing various auto-active ThsA SLOG-domain constructs. Leaf shown ~5 dpi, and all constructs infiltrated at OD 0.80. Framed numbers denote leaf replicates per set. Similar experiments were performed at least three times. (*D*) Anti-HA immunoblot of N-HA tagged ThsA WT or ThsA N112A performed in *Nb eds1$^{-/-}$* leaves.

SI 5

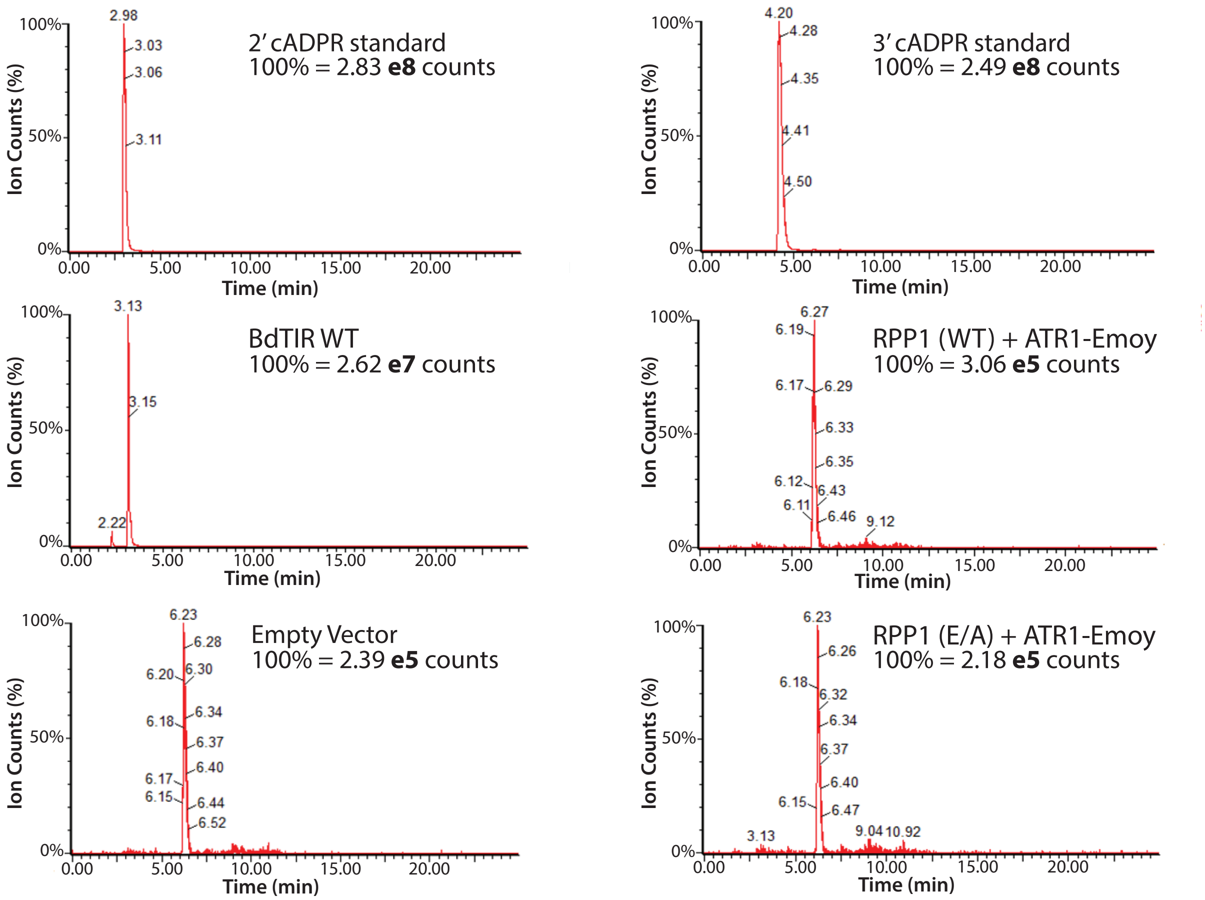

SI 5. Effector activated TIR-NLR RPP1 does not elevate v-cADPR (2’cADPR) *in planta* relative to plant BdTIR. LC-MS chromatograph traces of cADPR-isomers (MW 542) in *Nb eds1*$^{-/-}$ leaves transiently expressing RPP1_WsB + ATR1-Emoy, RPP1_WsB (E/A) + ATR1-Emoy, EV (35S:GFP, empty vector), BdTIR, or pure standards of 2’ and 3’cADPR (standards recently described in Manik et al (*14*). All constructs expressed at combined OD of 0.80. *Nb* leaves sampled at ~40 hpi.

SI 6

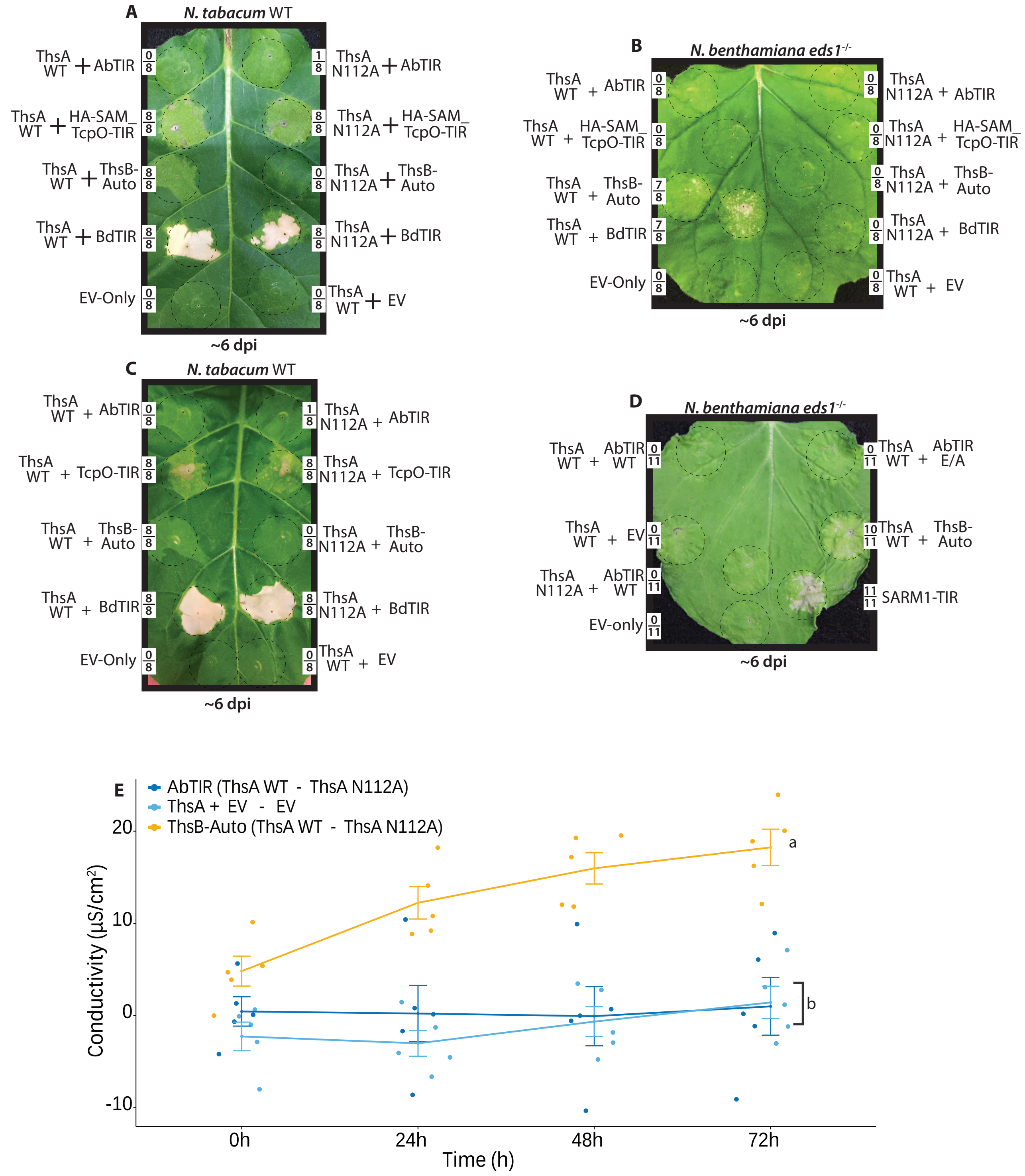

SI 6. Co-expression of AbTIR or TcpO-TIR does not enhance stimulation of ThsA activity relative to ThsB-Auto. (*A-D*) *N. tabacum* or *N. benthamiana eds1*$^{-/-}$ leaves co-expressing WT ThsA or N112A in combination with ThsB-Auto, or AbTIR or TcpO-TIR or EV (35S:GFP, empty vector). Framed numbers denote leaf replicates per set. The SAM-domain promotes oligomerization and refers to the sterile alpha motif of SARM1. ThsA and ThsB variants, or SARM1 or EV (35S:GFP) controls. ThsA N112A lacks SIR2-type NADase activity; ThsA R371A has an altered SLOG-motif, and ThsB E85Q lacks TIR-domain catalytic activity. All constructs expressed at OD 0.80. Relative to *N. benthaniama*, *N. tabacum* was consistently more susceptible to TcpO-TIR mediated cell death (EDS1-independent), even when expressed at the lower OD 0.40 used in co-expression studies. (*E*) Ion leakage assay in *Nb eds1*$^{-/-}$ leaves co-expressing different ThsA and ThsB combinations. Leaf discs were collected ~72 hpi, and ion measurements taken every 24 h for 3 days. Statistical analyses performed for final time point. High AbTIR expression alone (OD 0.80) elicits NAD^{+}-depletion and cytotoxicity to a limited degree (see Fig. 1), therefore, the difference of samples co-expressed with inactive ThsA N112A controls was plotted to reveal ThsA-stimulation. Similar experiments were performed at least three times. Statistical analyses: One-way ANOVA and Turkey HSD. Over-lapping letters are ns (non-significant) difference ($p>.05$) while separate letter class indicates $p<.05$ or better.

SI 7

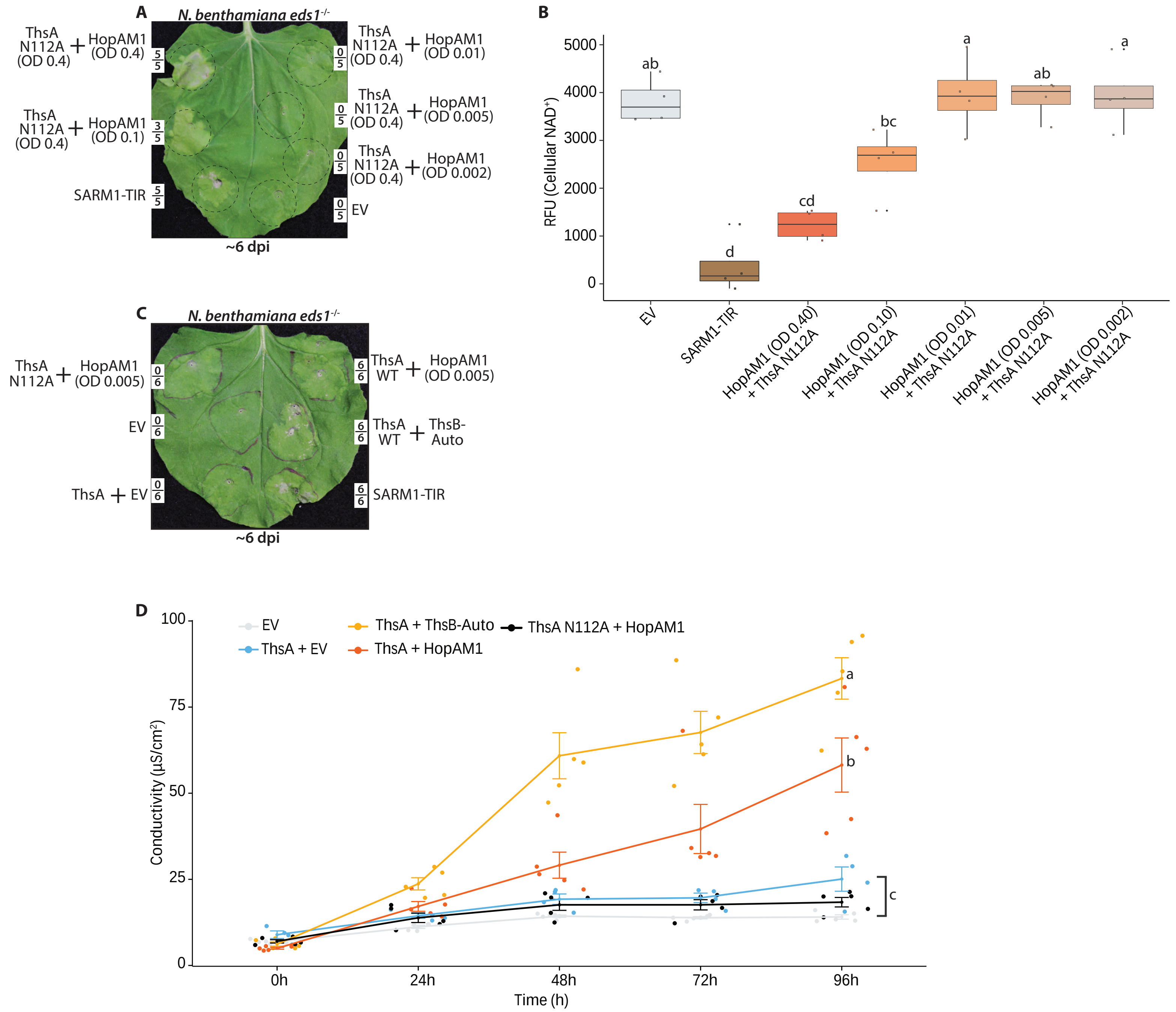

SI 7. HopAM1 causes dosage sensitive cell death independent of EDS1; non-cytotoxic doses of HopAM1 can stimulate ThsA-mediated cytotoxicity. (*A*) *Nb eds1*$^{-/-}$ leaves ~5 dpi with constructs expressing HopAM1 and ThsA N112A, or SARM1-TIR and EV (35S:GFP) controls. ThsA N112A lacks SIR2-type NADase activity. The delivered dosage of HopAM1 was titrated as noted on the leaf. All other constructs were expressed at OD 0.80. Framed numbers denote leaf replicates per set. (*B*) Fluorescent NAD$^+$-detection assay in *Nb eds1*$^{-/-}$ leaves co-expressing different HopAM1 dosages with inactive ThsA N112A. Leaves harvested ~40 hpi. (*C*) *Nb eds1*$^{-/-}$ leaves co-expressing WT ThsA or N112A in combination with ThsB-Auto, HopAM1, or EV (35S:GFP, empty vector). (*D*) Ion leakage assay in *Nb eds1*$^{-/-}$ leaves co-expressing ThsA, ThsB-Auto or HopAM1 combinations. Leaf discs were collected ~72 hpi, and measurements recorded every 24 h for 3 days. Similar experiments were performed at least three times. Statistical analyses performed for final time point. Statistical analyses: One-way ANOVA and Turkey HSD. Over-lapping letters are ns (non-significant) difference ($p>.05$) while separate letter class indicates $p<.05$ or better.

SI 8

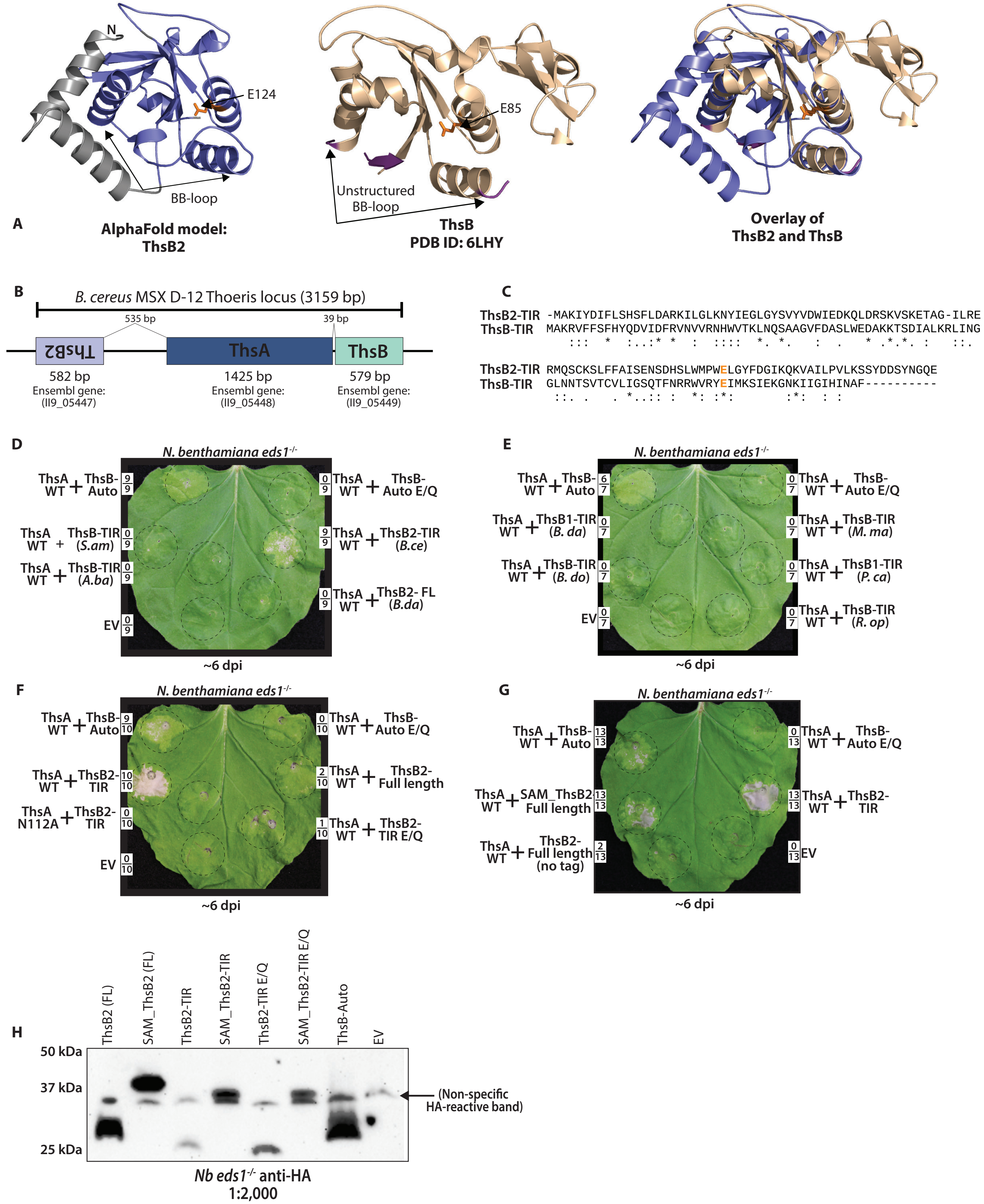

A
N
E124
BB-loop
AlphaFold model: ThsB2
E85
Unstructured BB-loop
ThsB PDB ID: 6LHY
Overlay of ThsB2 and ThsB
B
B. cereus MSX D-12 Thoeris locus (3159 bp)
535 bp
39 bp
ThsB2
ThsA
ThsB
582 bp Ensembl gene: (II9_05447)
1425 bp Ensembl gene: (II9_05448)
579 bp Ensembl gene: (II9_05449)
C
ThsB2-TIR -MAKIYDIFLSHSFLDARKILGLKNYIEGLGYSVYVDWIEDKQLDRSKVSKETAG-ILRE
ThsB-TIR MAKRVFFSFHYQDVIDFRVNVVRNHWVTKLNQSAAGVFDASLWEDAKKTSDIALKRLING
ThsB2-TIR RMQSCKSLFFAISENSDHSLWMPWELGYFDGIKQKVAILPVLKSSYDDSYNGQE
ThsB-TIR GLNNTSVTCVLIGSQTFNRRWVRYEIMKSIEKGNKIIGIHINAF----------
D
N. benthamiana eds1-/-
ThsA WT + ThsB-Auto 9/9
ThsA WT + ThsB-Auto E/Q 0/9
ThsA WT + ThsB-TIR (S.am) 0/9
ThsA WT + ThsB2-TIR (B.ce) 9/9
ThsA WT + ThsB-TIR (A.ba) 0/9
ThsA WT + ThsB2- FL (B.da) 0/9
EV 0/9
~6 dpi
E
N. benthamiana eds1-/-
ThsA WT + ThsB-Auto 6/7
ThsA WT + ThsB-Auto E/Q 0/7
ThsA WT + ThsB1-TIR (B. da) 0/7
ThsA WT + ThsB-TIR (M. ma) 0/7
ThsA WT + ThsB-TIR (B. do) 0/7
ThsA WT + ThsB1-TIR (P. ca) 0/7
EV 0/7
ThsA WT + ThsB-TIR (R. op) 0/7
~6 dpi
F
N. benthamiana eds1-/-
ThsA WT + ThsB-Auto 9/10
ThsA WT + ThsB-Auto E/Q 0/10
ThsA WT + ThsB2-TIR 10/10
ThsA WT + ThsB2-Full length 2/10
ThsA N112A + ThsB2-TIR 0/10
ThsA WT + ThsB2-TIR E/Q 1/10
EV 0/10
~6 dpi
G
N. benthamiana eds1-/-
ThsA WT + ThsB-Auto 13/13
ThsA WT + ThsB-Auto E/Q 0/13
ThsA WT + SAM_ThsB2 Full length 13/13
ThsA WT + ThsB2-TIR 13/13
ThsA WT + ThsB2-Full length (no tag) 2/13
EV 0/13
~6 dpi
H
ThsB2 (FL)
SAM_ThsB2 (FL)
ThsB2-TIR
SAM_ThsB2-TIR
ThsB2-TIR E/Q
SAM_ThsB2-TIR E/Q
ThsB-Auto
EV
50 kDa
37 kDa
25 kDa
(Non-specific HA-reactive band)
Nb eds1-/- anti-HA 1:2,000

SI 8

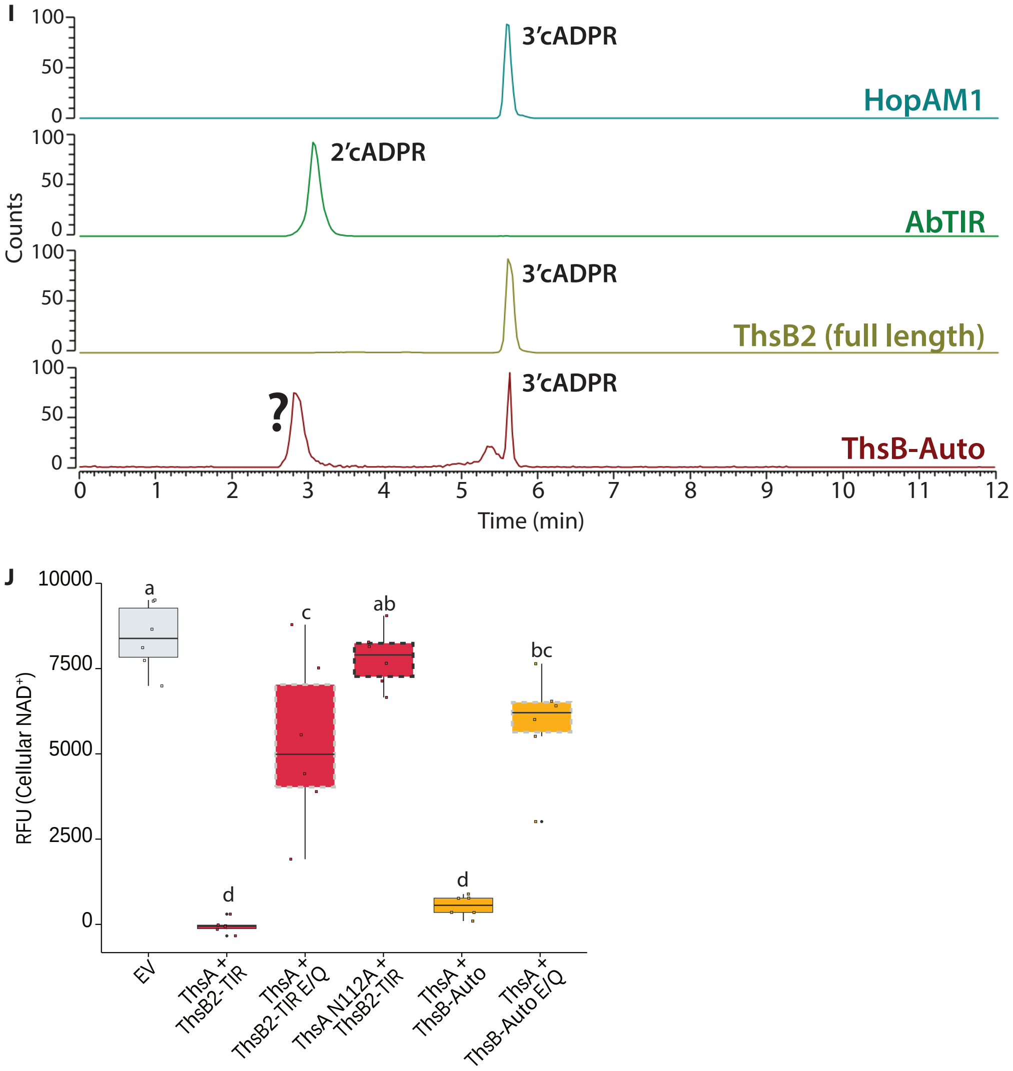

SI 8. The TIR-domain of a second ThsB encoded by *B. cereus* MSX-D12 is auto-active, produces 3'cADPR, and stimulates ThsA; no other tested ThsB-orthologs stimulated ThsA. (*A*) AlphaFold structure prediction of the second ThsB (ThsB2) from *B. cereus* MSX-D12 overlayed with the crystal structure of prototypical ThsB (PDB ID: 6LHY) of Ka *et al (33)*. (*B*) Schematic of the MSX-D12 Thoeris locus discovered by Doron *et al (43).* (*C*) ClustalW amino acid alignment of ThsB and ThsB2 reveals overall low shared residue identity. (*D-E*) *N. benthamiana eds1*$^{-/-}$ leaves expressing ThsA with ThsB-Auto, or the ThsB-orthologs screened in Fig S2. Framed numbers denote leaf replicates per set. (*F-G*) *N. benthamiana eds1*$^{-/-}$ leaves expressing ThsA with ThsB2-TIR (core TIR-domain) or full length ThsB2, as compared to ThsB-Auto. ThsB2-TIR alone does not cause cell death and catalytic E required to stimulate ThsA. (*H*) Anti-HA immunoblot of N-HA ThsB2-TIR or ThsB2 (FL, full length) proteins with or without SAM-oligomerization tag. E/Q: catalytic glutamate substitution. Proteins expressed in *Nb eds1*$^{-/-}$ and harvested ~30 hpi. (*I*) LC-MS analysis of *in vitro* NADase products from ThsB2, ThsB-Auto, and AbTIR or HopAM1. (*J*) Fluorescent NAD$^+$-detection assay in *Nb eds1*$^{-/-}$ leaves co-expressing ThsA WT (or ThsA N112A) with ThsB2-TIR or ThsB-Auto. E/Q substitutions in TIR-domain catalytic glutamate residue. Similar experiments were performed at least three times. Statistical analyses: One-way ANOVA and Turkey HSD. Over-lapping letters are ns (non-significant) difference (p>.05) while separate letter class indicates p<.05 or better.

SI 9

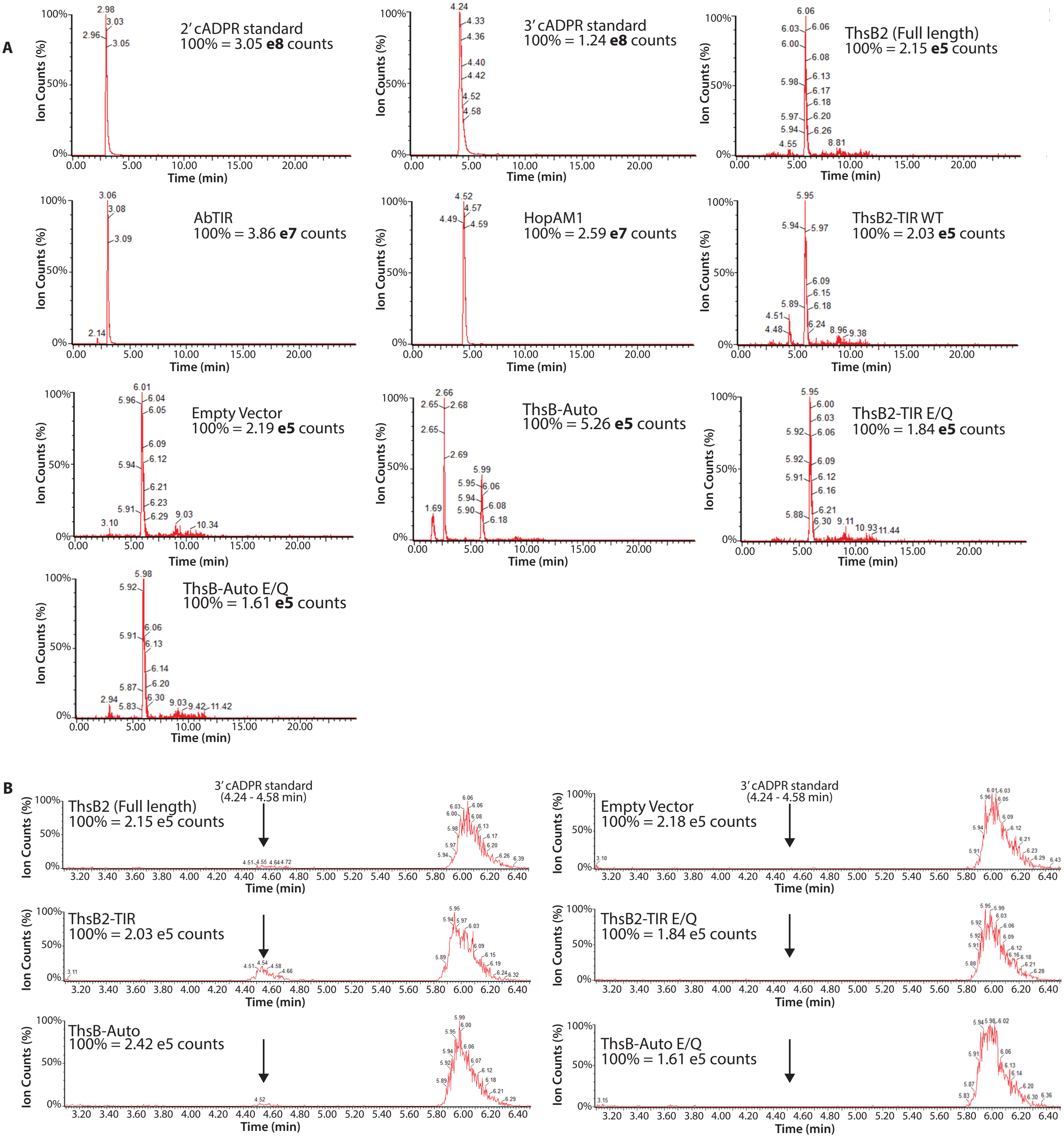

SI 9. ThsB-Auto and ThsB2 produce 3′cADPR *in planta*; TIR-domain catalytic glutamate is required. LC-MS chromatographs of cADPR-isomers (MW 542) from *Nb eds1*$^{-/-}$ leaves transiently expressing AbTIR, HopAM1, ThsB-Auto, ThsB2-TIR, ThsB2 (Full length), and empty vector (35S:GFP) or catalytic-null E/Q controls. Purified standards of 2′ and 3′cADPR were run simultaneously (*14*). All constructs infiltrated at OD 0.80; *Nb* leaves sampled ~40 hpi.

SI 10

TIR	Organism	Known enzymatic product(s)	EDS1-HR?	EDS1-independent cytotoxicity?	ThsA-activation?
**HopAM1**	*Pseudomonas syringae* (*DC3000*)	3'cADPR	X	✓	✓
**AbTIR**	*Acinetobacter baumanii*	2'cADPR	X	✓	X
**TcpO-TIR**	*Methanobrevibacter olleyae*	v-cADPR (2'cADPR?)	X	✓	X
**ThsB-Auto**	*Bacillus cereus* (*MSX D-12*)	3'cADPR, unknown isomer	X	X	✓
**ThsB2**	*Bacillus cereus* (*MSX D-12*)	3'cADPR	X	X	✓
**BdTIR**	*Brachypodium distachyon*	2'cADPR, 3'cADPR, pRib-AMP/ADP?, ADPr-ATP/ADP?	✓	X*	✓
**RPP1**	*Arabidopsis thaliana*	2'cADPR, 2',3'-cNMP, pRib-AMP/ADP, ADPr-ATP/ADP	✓	X	X
**SARM1-TIR**	*Homo sapiens*	cADPR, ADPR	X	✓	Not examined** Ofir *et al*, ref. (*16*)

SI 10. Summary of examined TIR-domains: reported enzymatic products and EDS1 / ThsA-signaling phenotypes. Orange background indicates TIR-proteins of prokaryotic origin; green indicates plant TIRs, and grey indicates human SARM1-TIR. EDS1-independent cytotoxicity in *Nicotiana benthamiana* by AbTIR and TcpO-TIR is dosage sensitive, correlates with NAD^+-depletion, and typically requires prolonged over-expression of OD_{600} >0.40. *BdTIR can also elicit EDS1-independent toxicity under specific contexts such as prolonged over-expression using Omega translational enhancers. **SARM1 not examined as canonical cADPR was previously found to not stimulate ThsA in Ofir *et al* (*16*).

Supplementary Data

Dataset-S1. Cloned/synthesized genes and oligonucleotide primers

Dataset-S2. Data for main and supplemental figures
